# Mesoporous LiMn_1–x_M_x_PO_4_ (M = Ni, Co) Precatalysts by Alcohol-Mediated Self-Assembly: Hydroxide Reconstruction and Suppression of Manganese Degradation for Stable Alkaline Oxygen Evolution

**DOI:** 10.1021/acs.inorgchem.6c03469

**Published:** 2026-08-18

**Authors:** Arda Altan, Irmak Karakaya Durukan, Ömer Dag

**Affiliations:** † Department of Chemistry, 52948 Bilkent University, Ankara 06800, Türkiye; ‡ UNAM - National Nanotechnology Research Center and Institute of Materials Science and Nanotechnology, Bilkent University, Ankara 06800, Türkiye

## Abstract

Manganese is an earth-abundant, low-cost transition metal with considerable potential for sustainable electrocatalysis; however, its poor stability under oxidative alkaline conditions remains a major challenge. Herein, we report the fabrication of Ni­(II)- and Co­(II)-substituted olivine lithium manganese phosphate electrodes, LiMn_1–x_M_x_PO_4_ (M = Ni, Co; x = 0.05, 0.10, and 0.25), through an alcohol-mediated synthesis approach and investigate the effects of solvent and secondary metal incorporation on their structural evolution and alkaline oxygen evolution reaction (OER) performance. The precursor solutions exhibit markedly different precipitation behaviors depending on the solvent. Rapid precipitation of crystalline MnPO_4_·H_2_O occurs in ethanol and 1-butanol, whereas precipitation is slower and less extensive in methanol. Increasing Ni­(II) concentration suppresses precipitation more effectively than Co­(II), improving the stabilization of Mn­(II) in solution. Upon calcination, ethanol- and 1-butanol-derived precursors yield predominantly mesoporous olivine LiMn_1–x_M_x_PO_4_ with minor Mn_2_P_2_O_7_ impurities, whereas methanol-derived solutions and supernatants produce phase-pure olivine LiMn_1–x_M_x_PO_4_. Electrochemical studies in 1 M KOH reveal rapid reconstruction of all phosphate electrodes into mixed-metal hydroxides, identifying these hydroxides as the active OER species. Secondary metal incorporation substantially enhances electrode stability by suppressing manganese degradation and promoting stable mixed (Mn,M)­(OH)_2_ active phases.

## Graphical abstract

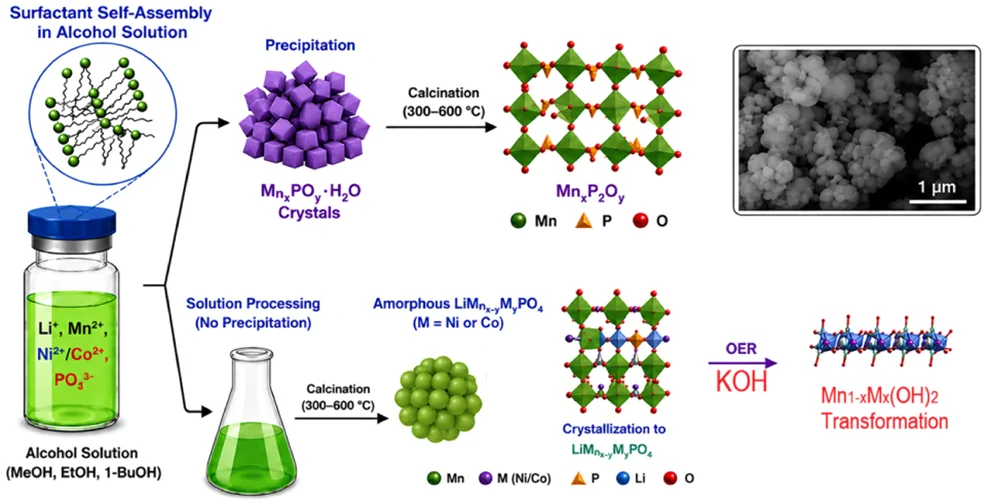

## Introduction

Transition metal based-materials constitute an important class of materials for clean energy generation and storage.^1–7^ In particular, mesoporous electrodes have attracted significant attention for applications in electrocatalysis, supercapacitors, and rechargeable batteries owing to their high surface areas, tunable pore architectures, and efficient mass transport properties.^8–12^ Among these applications, water electrolysis has emerged as a promising technology for sustainable green hydrogen production through the splitting of water into hydrogen and oxygen.^13–17^ Water electrolysis relies on two electrochemical half-reactions: the hydrogen evolution reaction (HER) at the cathode and the oxygen evolution reaction (OER) at the anode. Of these two processes, OER is kinetically more demanding and therefore represents the major bottleneck in overall water-splitting efficiency. Consequently, there is a strong demand for scalable, processable, stable, and earth-abundant electrode materials capable of efficiently catalyzing OER. In this context, mesoporous materials are attractive because their high accessible surface areas and processability of the process maximize the number of active sites available for electrochemical reactions.

Various approaches have been developed to synthesize mesoporous electrode materials, including surfactant-directed sol–gel methods and hard-templating strategies, followed by fabrication into electrodes using suitable binders.^18–22^ Alternatively, the molten salt-assisted self-assembly (MASA) method offers a versatile route for directly fabricating mesoporous thin-film electrodes.^23–28^ In this approach, homogeneous solutions containing metal salts, acids, and surfactants are deposited onto conductive substrates, where they self-assemble into lyotropic liquid crystalline mesophases that can subsequently be converted into mesoporous inorganic thin films through thermal treatment.^23–28^ This strategy enables direct thin-film electrode fabrication while maintaining high surface areas and controlled pore structures.

Transition-metal phosphates and pyrophosphates represent an important family of electrode materials^29–32^ that can also be synthesized using modified MASA approaches.^33–36^ These compounds are particularly attractive because transition-metal ions can act both as structural components and as active centers for electrochemical reactions. In addition to the choice of metal ion, the precursor chemistry and solvent environment play critical roles in controlling self-assembly, phase formation, morphology, and porosity.^33–38^ Among the earth-abundant transition metals, iron and manganese are especially attractive due to their low cost, natural abundance, and rich redox chemistry. However, developing stable and highly active manganese- and iron-based phosphates or pyrophosphates remains challenging.^39–42^ Therefore, understanding the role of solvents in the synthesis and processing of these materials is essential for the rational design of mesoporous electrode architectures.

One of the major challenges associated with manganese- and iron-based OER electrocatalysts is the instability of their high-valent oxidation states under alkaline conditions.^43–46^ To achieve efficient water oxidation, the metal must be oxidized to high oxidation states that can participate in O–O bond formation.^27,28^ However, these oxidized species are susceptible to disproportionation reactions, producing soluble FeO_4_^2-^ and MnO_4_^–^ species that result in catalyst degradation and loss of activity.^43^ In the case of manganese, Mn­(VI) species can undergo disproportionation to form soluble Mn­(VII) and MnO_2_ products, leading to dissolution of the active material.^43^ Modifying the local chemical environment around manganese can suppress this degradation pathway and stabilize the high-valent manganese species.^27,28^ One effective strategy is the incorporation of secondary transition metals such as Co, Ni, or Cu. These metals possess higher electronegativities and can actively participate in OER at lower oxidation states, thereby enhancing catalytic performance while improving structural stability.^27^

Recently, we employed the MASA approach to investigate the role of alcohols as solvents in the synthesis of mesoporous transition-metal phosphates and pyrophosphates.^35–38^ Replacing water with alcohols significantly alters the coordination chemistry and self-assembly behavior of the metal–phosphate/pyrophosphate systems, enabling the formation of high-surface-area mesoporous materials.^36–38^ Building on these findings, the present study explores the combined effects of solvent-mediated self-assembly and secondary-metal incorporation on the synthesis of stable manganese-based electrode materials. Nickel and cobalt were introduced into manganese phosphate systems at concentrations of 5, 10, and 25 mol %, and the resulting mesoporous electrode materials were evaluated as OER electrocatalysts in alkaline media. Particular attention was given to their structural evolution and stability, as these phosphate-based electrodes undergo *in situ* transformation into mixed-metal hydroxide phases under alkaline conditions. The relationship between solvent choice, composition, structure, phase transformation, and electrocatalytic performance is systematically investigated to establish design principles for stable and efficient manganese-based OER electrodes.

## Experimental Section

### Materials

All salts (LiNO_3_), manganese­(II) nitrate tetrahydrate ([Mn­(H_2_O)_4_]­(NO_3_)_2_), nickel­(II) nitrate hexahydrate ([Ni­(H_2_O)_6_]­(NO_3_)_2_, and cobalt­(II) nitrate hexahydrate ([Co­(H_2_O)_6_]­(NO_3_)_2_)), solvents (methanol (99.9%), ethanol (99.9%), and 1-butanol (99.0%)), surfactant (Pluronic P123 (EO_20_–PO_70_–EO_20_, *M*_n_ ≈ 5800 g mol^–1^)) and acid (phosphoric acid (85–88 wt %)) were purchased from Sigma–Aldrich and used without further purification.

### Preparation of Salts–Phosphoric Acid–P123 Solutions

Solutions were prepared in two different 25 mL glass vials (acid solution and salt solution) using a fixed P123:LiNO_3_:M­(NO_3_)_2_ (M is Mn + Ni or Co):H_3_PO_4_ molar ratio of 1:30:30:30 in 10 g of solvent. The exact quantities are listed in Table S1.

Dissolve lithium nitrate (0.357 g) in 5 g of solvent in the first vial (2–3 min), then add metal nitrates and stir the mixture for another 3–5 min. Dissolve phosphoric acid in another 5 g of solvent in the second vial. Add 0.5 g premelted P123 (80 °C) to each vial and stir for an additional 5 min. Once homogeneous, pour the acid solution into the salt-containing vial under stirring. This mixing created immediate precipitations in both ethanol and 1-butanol solutions. Methanol solution is stable for a longer duration and give small amount of precipitate.

### Preparation of Gel-Phases and Mesoporous Materials

Separate the precipitate from the rest of the solution by centrifugation, wash twice with the corresponding solvent, and dry under ambient laboratory conditions for 24 h to obtain the precipitates. The solution (mixture without separation of the precipitate fraction) and supernatant fractions were processed separately. Both fractions were deposited either by drop-casting onto glass slides or by pouring into Pyrex Petri dishes, followed by aging at room temperature (1–2 days) to form thin gel films or bulk gels, respectively. The structural evolution at room temperature is monitored by ATR-FTIR and XRD using either coated glasses or dropped solutions.

Dried gels or precipitates were first calcined at 300 °C for 1 h and subsequently transferred to ceramic crucibles for further heat treatments in 100 °C increments up to 700 °C, thereby avoiding reactions between the samples and glass substrates.

### Preparation of FTO and Graphite Rod Coated Electrodes

Electrodes were prepared on fluorine-doped tin oxide (FTO) glass and graphite rods (GRs) using solutions or supernatants for electrochemical measurements (see Figure S1). FTO substrates (2 × 1 cm^2^) were masked to expose a 1 × 1 cm^2^ active area and coated by spin coating at 5000 rpm for 10 s. After removing the tape, the electrodes were calcined at 300 °C and subsequently annealed up to 500 °C. Graphite rods (15 cm length) were cut into 3 cm segments, polished using a figure-eight motion to ensure uniform coatings and reproducibility, washed with ethanol, and dried at 80 °C for 24 h. The rods were dip-coated to obtain a 1 cm^2^ active area using solutions or supernatants diluted 5-fold with the corresponding solvent to control film thickness. The coated GR electrodes were calcined at 300 °C for 1 h and further annealed at 400 and 500 °C.

## Instrumentation

### Small-Angle X-ray Diffraction (XRD)

Small-angle (1–5°, 2θ) XRD patterns of the drop-cast solution and supernatant samples were recorded using a Rigaku Miniflex diffractometer equipped with a Cu Kα radiation source (λ = 1.5405 Å), operated at 30 kV and 15 mA, with a NaI­(Tl) detector and Be window. The scan speed was 0.5° min^–1^ with a step size of 0.01°.

### Wide-Angle X-ray Diffraction (XRD)

Wide-angle (10–80°, 2θ) XRD patterns of the powder samples were collected using either a Rigaku Miniflex and/or a PANalytical multipurpose diffractometer equipped with a Cu Kα source (λ = 1.5405 Å) operated at 45 kV and 40 mA.

### Attenuated Total Reflectance Fourier Transform Infrared (ATR-FTIR) Spectroscopy

ATR-FTIR spectra were recorded in the 400–4000 cm^–1^ range at a resolution of 4 cm^–1^ with 32 scans using a Bruker Alpha Platinum spectrometer equipped with a DigiTect DLATGS detector. A few drops of liquid samples were placed directly on the ATR crystal, while small amounts of gel-like precipitate or powder samples were similarly analyzed to monitor gel formation and structural features. Powder samples were directly put on ATR crystal and pressed to measure their ATR-FTIR spectra.

### Scanning Electron Microscopy (SEM)

SEM images of the powder samples were obtained using an FEI Quanta 200 FEG microscope operated at 15 kV with a 3 nm spot size under a pressure of ∼10^–6^ Torr. Samples were mounted on aluminum stubs using carbon tape.

### N_2_ Adsorption–Desorption Analysis

N_2_ adsorption–desorption isotherms were recorded at 77.4 K using a Micromeritics TriStar 3000 automated gas adsorption analyzer over a relative pressure (P/P_0_) range of 0.02–0.99 with 5 min equilibration intervals. Sample tubes were cleaned with aqua regia, rinsed thoroughly with deionized water and ethanol, and dried for 30 min. Approximately 150 mg of sample powder was loaded after evacuation under vacuum for 1 h, followed by degassing at 200 °C under 50 mTorr for 2 h. The samples were then weighed and placed in an isothermal jacket for measurement.

### X-ray Photoelectron Spectroscopy (XPS)

XPS measurements were performed using a Thermo Scientific Kα spectrometer equipped with a monochromatic Al K_α_ source (1486.68 eV). Powder samples were mounted on copper tape and analyzed under ultrahigh vacuum using a 400 μm spot size. Binding energies were calibrated using the C 1s peak at 285.0 eV.

### Electrochemical Measurements

Electrochemical characterizations were performed using a three-electrode setup with a platinum wire counter electrode, an Ag/AgCl (3.5 M KCl) reference electrode, and the graphite rod (GR), coated with the samples as working electrodes in 1 M KOH electrolyte. A Gamry potentiostat (IFC5000–07565) was employed for all measurements, and potentials were referenced to the normal hydrogen electrode (NHE). Cyclic voltammetry (CV) was conducted between −0.5 and 1.5 V for GR and −0.5 and 2.0 V for FTO coated electrodes at a scan rate of 50 mV s^–1^. Tafel slopes were derived from chronoamperometric measurements performed between 0.5 and 0.8 V with 10 mV increments (300 s per step). Electrode stability was evaluated by chronopotentiometry at current densities ranging from 1 mA cm^–2^ to 100 mA cm^–2^ (10 mA cm^–2^ increments) for 60 min per step.

## Results and Discussion

### Solution and Gelation Behavior of Li­(I)–Mn­(II)–Ni­(II)/Co­(II)-PA-P123 Mixtures

Alcohol-based precursor solutions were prepared by first making two separate solutions: Solution 1 contained the transition metal nitrates (Mn­(II)/Ni­(II) or Mn­(II)/Co­(II)), lithium nitrate, and P123, while Solution 2 contained phosphoric acid (PA) and P123. Note that each solution forms a lyotropic liquid crystalline phases upon solvent evaporation.^23,25,44^ After homogenization, the two solutions were mixed to obtain the final precursor solutions. The molar composition of the mixtures was maintained at 30LiNO_3_:30­[(1–x)­Mn­(NO_3_)_2_·4H_2_O + xM­(NO_3_)_2_·nH_2_O]:30PA:1P123, where M = Ni­(II) or Co­(II). Ni­(II) or Co­(II) was introduced into the Mn­(II) system at molar fractions of 5, 10, and 25%, while the Mn­(II) content was correspondingly reduced to 95, 90, and 75%, respectively. The samples are denoted as LMnMXP-P123, where M represents the secondary metal (Ni or Co) and X corresponds to its molar percentage (5, 10, or 25%).

Upon mixing, the precursor solutions underwent precipitation and/or gelation, depending on the solvent (methanol, ethanol, or 1-butanol) system. The precipitated fractions were separated to obtain the corresponding supernatants, and both the original solutions and the supernatants were transferred into Pyrex Petri dishes and left for approximately 1 day to allow solvent evaporation. In the 1-butanol system, gelation was observed during solvent evaporation, which required 2–3 days to complete. The resulting gel phases were subsequently heated at 100 °C for 1 h to completely remove the residual solvent. The dried gels were then calcined at 300 °C. For further thermal treatment, the calcined materials were transferred to ceramic crucibles and annealed at higher temperatures. These samples are denoted as LMnMXP-Y, where L is lithium, M is the secondary transition metal, X is its molar percentage in the final product, P is phosphate, and Y is the calcination/annealing temperature (°C).

To investigate the gelation process, a drop of either the precursor solution or the supernatant (when precipitation was minimal) was placed on the ATR-FTIR diamond crystal and monitored for 24 h. Similarly, drop-cast films prepared on glass substrates were used to follow structural evolution by XRD. Because the mixed-metal systems contain a significantly higher concentration of Mn­(II) than Ni­(II) or Co­(II), precipitation occurs in all solvent systems.^36^ The precipitation was most rapid in 1-butanol, followed by ethanol, whereas the slowest precipitation was observed in methanol. Furthermore, the amount of precipitate formed in the Ni-containing systems was lower than that observed in the Co-containing systems, which may be related to differences in the nucleation and growth behavior of the initially formed nanostructures. The precipitates were collected by centrifugation and washed with the corresponding solvent.

After complete drying, the precipitates were characterized by wide-angle XRD and ATR-FTIR spectroscopy. It has previously been established that pure Mn­(II)–phosphate solutions are inherently unstable and gradually produce crystalline precipitates.^36^ Figure 1a displays the XRD patterns, obtained from all solvent systems (ethanol, methanol, and 1-butanol) at 5 and 25% Ni ethanol solutions. The XRD patterns can be indexed to monoclinic MnPO_4_·H_2_O with the *C*2/*c* space group. The dried/washed precipitates were further analyzed by ATR-FTIR spectroscopy to confirm their phase purity. The spectra exhibit the characteristic vibrational fingerprint of MnPO_4_·H_2_O crystals and show no evidence of residual surfactant or nitrate species in the precipitates (Figure 1b). Although the phosphate vibrational bands are nearly identical in all spectra, the crystalline-water stretching band centered at approximately 3100 cm^–1^ is significantly sharper for precipitates formed in ethanol and 1-butanol than for those obtained from methanol. This difference suggests a higher degree of crystallinity and/or larger crystallite size in the ethanol- and 1-butanol-derived precipitates, whereas the broader water band in the methanol-derived sample indicates the formation of smaller and more structurally disordered crystallites.

**Figure 1:**
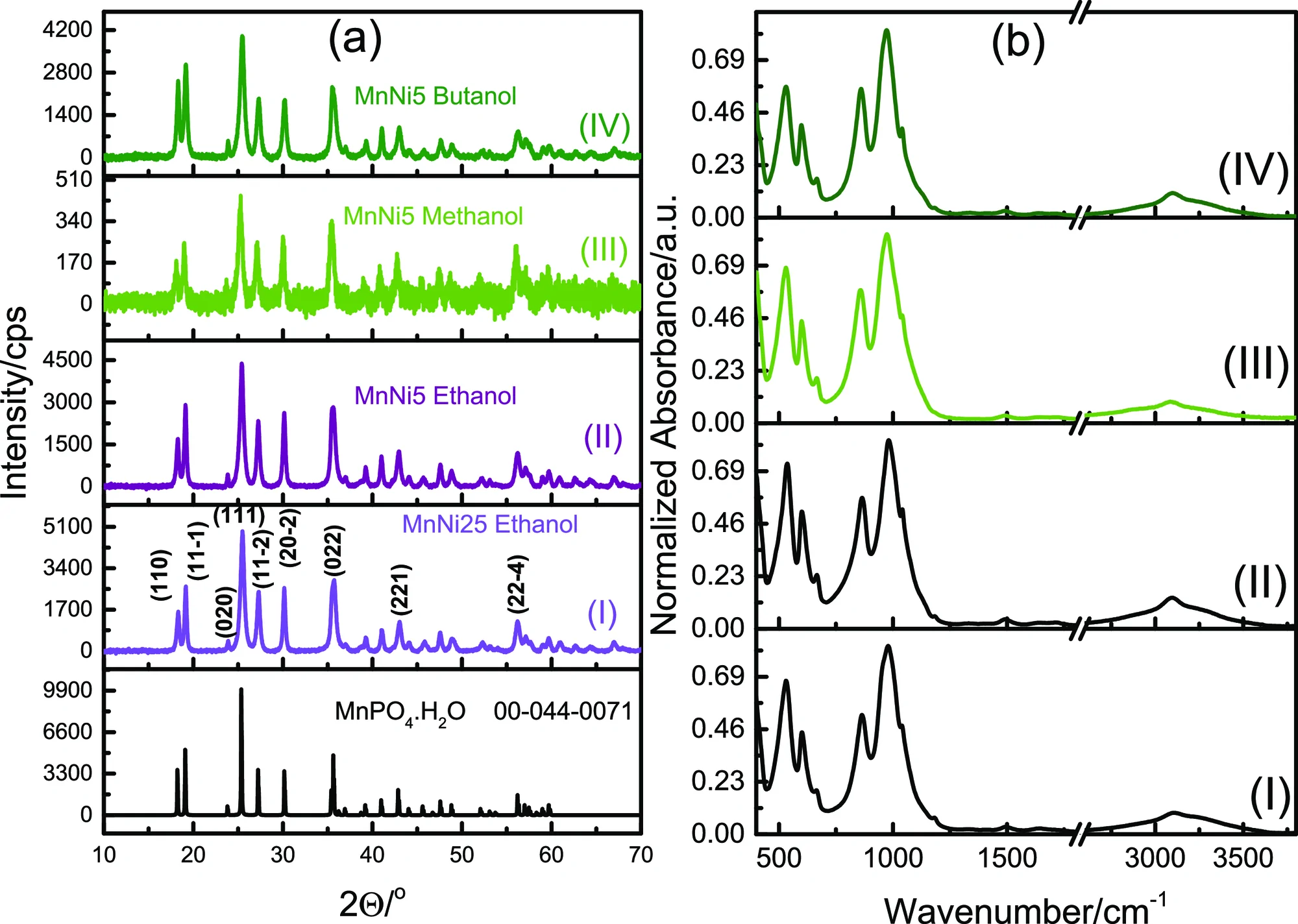
(I) Ethanol 25% Ni, (II) ethanol 5%, (III) methanol 5%, and (IV) 1-butanol 5% LMnNiXP-P123 precipitates: (a) Wide-angle XRD patterns with the reference pattern of MnPO_4_·H_2_O and (b) ATR-FTIR spectra.

The precipitation alters the composition of the remaining solution by increasing the Li­(I) amount and Ni­(II)/Mn­(II) or Co­(II)/Mn­(II) molar ratios in the supernatant fractions relative to the initial precursor mixtures. Consequently, the gel phases formed from the supernatants become enriched in Li­(I) and Ni­(II) or Co­(II), respectively. To further evaluate the stability and structural evolution of the mesophases during gelation at room temperature (RT), both the supernatants and the original solutions were characterized by small-angle XRD. Figure 2 displays the small-angle XRD patterns of the ethanol- and methanol-derived LMnNi5P–P123 supernatants at RT. The mesophase forms almost immediately upon spreading the ethanol-derived supernatant on a glass slide. Within 10 min, two diffraction lines appear at 1.39 and 2.13° (2θ), characteristic of an ordered mesophase (Figure 2a). Upon further aging, these reflections gradually shift to higher angles, indicating contraction of the mesostructure during solvent evaporation. Nevertheless, at least one diffraction line remains detectable even after 1 day of aging, suggesting partial retention of mesoscopic order. Prolonged aging, however, ultimately leads to the formation of a more disordered phase. In contrast, the methanol-derived supernatant exhibits only weak diffraction features during the first 10 min, which subsequently disappear as the sample becomes completely disordered (Figure 2b). These findings suggest that mesophase formation is governed by a delicate balance between solvent evaporation kinetics and precursor composition. The slower evaporation rate of ethanol, together with the higher concentrations of Li­(I), lower concentration of the transition-metal ions, and phosphoric acid species in the supernatant, facilitates cooperative self-assembly and transient mesophase ordering, whereas the methanol system lacks sufficient driving force to maintain long-range mesoscopic order. Moreover, increasing Ni to 25% alters the gelation process slightly, where the diffraction lines shift to lower angles with solvent evaporation and further reaction in the gel phase (Figure 2c).

**Figure 2:**
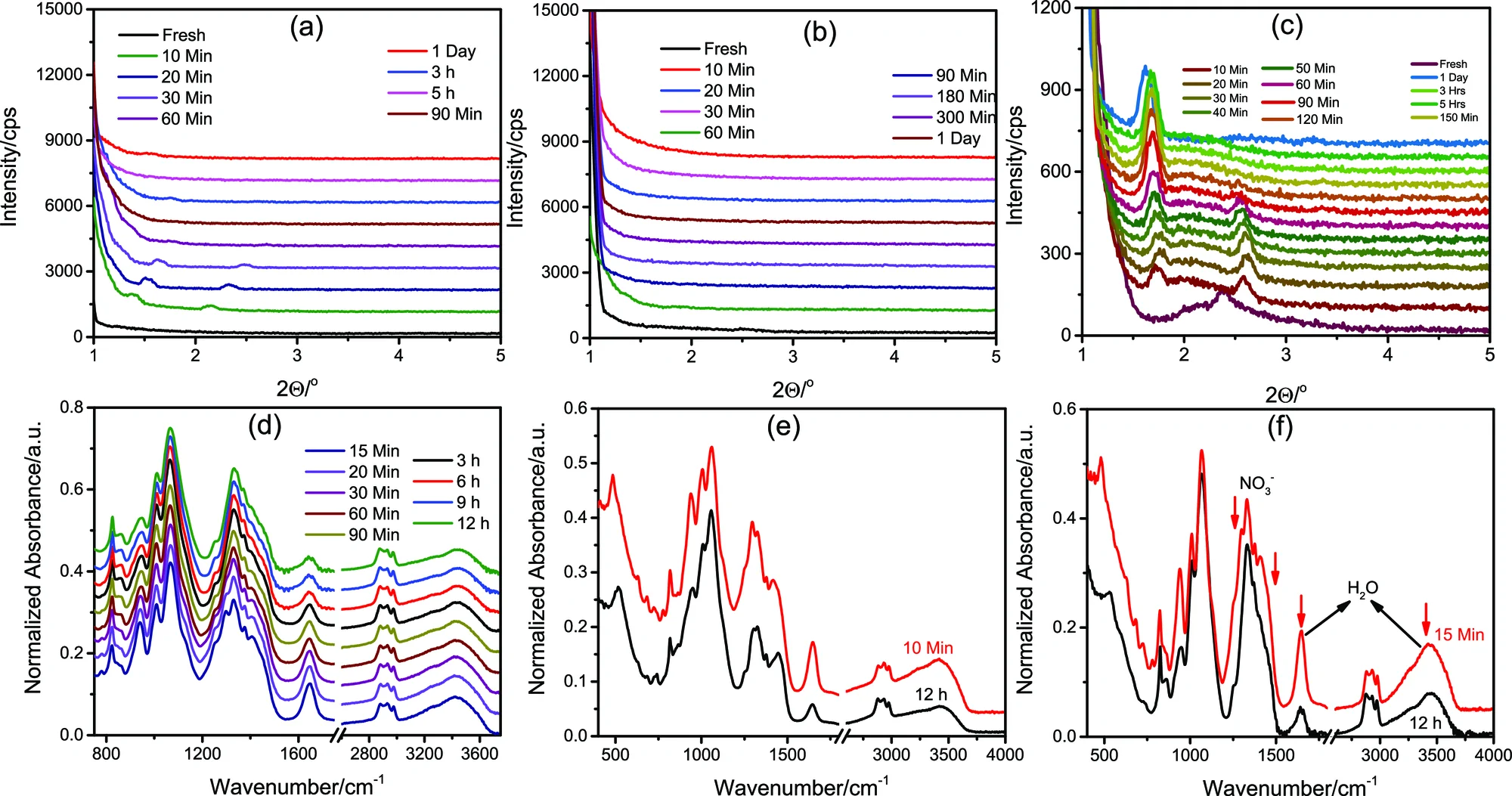
Small-angle XRD patterns: the LMnNi5P from supernatants of (a) ethanol and (b) methanol, and LMnNi25P (c) ethanol during gelation. ATR-FTIR spectra: LMnNi5P from (d) ethanol and (e) methanol supernatants, and (f) LMnNi25P ethanol supernatant (during gelation).

Figure 2d displays the ATR-FTIR spectral evolution during the aging/gelation of the ethanol-derived supernatant. The spectra exhibit intense nitrate bands in the 1300–1500 cm^–1^ region, arising from unreacted metal nitrate and lithium nitrate species retained in the gel phase. During the first 10 min, the spectra are dominated by the characteristic vibrational features of ethanol. However, after approximately 15 min, the ethanol bands completely disappear, indicating that gelation and solvent evaporation are essentially complete. Upon further aging, some changes are observed in the nitrate and phosphate regions, whereas a gradual decrease in the intensity of the water bands is evident, including the H–O–H bending mode at 1640 cm^–1^ and the broad O–H stretching band centered at approximately 3500 cm^–1^. Overall, the ATR-FTIR spectra exhibit only subtle variations over 12 h of aging, suggesting that the chemical composition of the gel phase remains largely unchanged after gelation, with the primary change being the gradual loss of residual water. During gelation, the nitrate bands progressively decrease in intensity, indicating the partial removal and/or decomposition of nitrate species as nitric acid and/or its decomposition products. Simultaneously, the phosphate vibrational bands become more pronounced, while the water-related bands decrease in intensity. These spectral changes are consistent with the ongoing condensation and polymerization reactions occurring within the gel phase and provide strong evidence for the chemical transformation described by eq 1.

1 $$nLiNO_{3}+\left(n-x\right)Mn{\left(NO_{3}\right)}_{2}/xM{\left(NO_{3}\right)}_{2}+nPA+P123\to (\mathrm{LiM}n_{1-x}M_{x}H_{y}PO_{4}{\left(NO_{3}\right)}_{y}{)}_{n}‐P123\left(gel\right)+\left(3n-ny\right)HNO_{3}\left(g\right)$$

In contrast, precipitation is significantly less pronounced in methanol; consequently, the composition of the supernatant remains much closer to that of the initial precursor solution. The ATR-FTIR spectra of the methanol-derived supernatant are shown in Figure 2e. After complete evaporation of methanol, the nitrate-to-phosphate peak intensity ratio differs markedly from that observed for the ethanol-derived supernatant. Specifically, the intensity ratio of the nitrate band at 1305 cm^–1^ to the phosphate band at 1108 cm^–1^ decreases from 0.67 to 0.40 during 12 h of aging (Figure 2e). This behavior suggests that the reaction described in eq 1 proceeds to a greater extent in the methanol system. Consequently, a larger fraction of the nitrate ions becomes coordinated to the metal centers, leading to the splitting of the asymmetric nitrate stretching band into two components in the 1300–1500 cm^–1^ region. The stronger metal–nitrate interactions and the resulting distribution of nitrate coordination environments introduce a higher degree of disorder into the gel phase, consistent with the loss of long-range mesoscopic order observed in the small-angle XRD patterns. Similar trends are observed in the other two compositions (LMnNi10P and LMnNi25P), see Figures 2f and S2.

The supernatants, obtained from the Co­(II)/Mn­(II) systems, were not investigated in detail because extensive precipitation substantially changes the solution composition. Therefore, only the original solutions and precipitates were examined for the ethanol- and 1-butanol-based LMnCoXP mixtures. The methanol-derived LMnCoXP supernatants exhibited behavior analogous to that of the corresponding LMnNiXP methanol supernatants and were discussed together.

In contrast, methanol-derived supernatants yielded the desired mesoporous olivine phases upon gelation/calcination. Because a small precipitation of MnPO_4_·H_2_O phase enriches the supernatant in Li­(I) species and modifies the Li­(I)/Mn­(II) ratio to favor o-LiMn_1–x_M_x_PO_4_ formation. Consequently, the ethanol- and 1-butanol-derived systems were generally analyzed as whole precursor mixtures without separating the precipitated MnPO_4_·H_2_O whenever precipitation was significant. Because precipitation is less extensive and proceeds more slowly in methanol than in ethanol or 1-butanol, no diffraction lines attributable to crystalline MnPO_4_·H_2_O are detected in the wide-angle XRD patterns of the methanol-derived solution phase (Figure S3). Therefore, the supernatant and the solution were analyzed separately for the methanol-based mixtures.

Mechanistically, increasing the concentration of Ni^2+^ or Co^2+^ while decreasing the Mn^2+^ content suppresses the oxidation of Mn^2+^ during the synthesis. The extent of MnPO_4_·H_2_O formation is strongly dependent on the alcohol used and is most pronounced in 1-butanol. Since the alcohol is the only available oxidizing agent in the reaction medium, its oxidizing power is insufficient to oxidize the added Ni^2+^ or Co^2+^ species. Furthermore, the Mn^2+^/Mn^3+^ redox process is likely governed by an equilibrium that is more favorable in alcohol-rich media. The addition of Ni^2+^ or Co^2+^ also increases both the water content and the acidity of the reaction medium because Ni­(NO_3_)_2_·6H_2_O or Co­(NO_3_)_2_·6H_2_O introduces six coordinated water molecules per metal ion into the solution. The increased hydration and acidity stabilize Mn^2+^, thereby shifting the redox equilibrium toward the reduced state and suppressing its oxidation to Mn^3+^. Consequently, the formation of MnPO_4_·H_2_O decreases with increasing Ni^2+^ or Co^2+^ content. Moreover, the incorporation of Ni^2+^ or Co^2+^ accelerates the gelation process. The faster formation of the gel network kinetically suppresses the relatively slow oxidation of Mn^2+^ and the subsequent crystallization of MnPO_4_·H_2_O, further reducing the extent of this side reaction.

### Mesoporous LiMn_1–x_Ni_x_PO_4_ (x = 0.05, 0.10, and 0.25)

In all binary-metal compositions investigated (5, 10, and 25 mol % Ni), the initially formed crystalline MnPO_4_·H_2_O precipitate undergoes thermal transformation to Mn_2_P_2_O_7_ upon calcination.^36,47,48^ The ethanol- and 1-butanol-derived systems were generally analyzed as whole precursor mixtures without separating the MnPO_4_·H_2_O crystals. In contrast, methanol-derived supernatants yielded the desired mesoporous olivine phase, likely because the precipitation of MnPO_4_·H_2_O is much smaller and modifies the Li­(I)/Mn­(II) ratio to favor o-LiMn_1–x_Ni_x_PO_4_ formation.

The ATR-FTIR spectra and wide-angle XRD patterns of the precipitates obtained from the Ni­(II)/Mn­(II) mixtures in ethanol and calcined at various temperatures are presented in Figure 3. Both techniques clearly demonstrate the progressive conversion of MnPO_4_·H_2_O into Mn_2_P_2_O_7_ upon heating similar to the pure Mn­(II) system.^36^ The transformation becomes particularly evident at 400 °C, where the characteristic vibrational bands and diffraction lines of MnPO_4_·H_2_O disappear and are replaced by those corresponding to crystalline beta-Mn_2_P_2_O_7_. These results confirm that the precipitated manganese phosphate hydrate serves as the precursor phase for the formation of manganese pyrophosphate during calcination in the solution-derived samples. Because precipitation significantly alters the Li­(I)/Mn­(II) ratio in the remaining solution and is much more extensive in ethanol and 1-butanol than in methanol, the desired olivine LiMn_1–x_Ni_x_PO_4_ (abbreviated as MnNiX-Y, to distinguish calcined samples from gel-samples, where MnNi represent the olivine structure, X is Ni mole percent, and Y is calcination temperature in Celsius) phase could not be obtained from the ethanol- and 1-butanol-derived systems. Figure 3c presents the ATR-FTIR spectra of the RT and calcined samples obtained from the ethanol supernatants. In all compositions, characteristic nitrate/carbonate bands are observed in the 1300–1500 cm^–1^ region. However, the intensity of these bands decreases systematically with increasing Ni­(II) content, as illustrated in Figure S4a–c. The supernatant fractions are consistently enriched in Li­(I) due to the preferential precipitation of Mn as MnPO_4_·H_2_O. Consequently, Li_3_PO_4_ is the predominant crystalline phase formed upon calcination at 300 °C. In addition to Li_3_PO_4_, NaNO_3_ crystals are also detected in the calcined products,^36^ as confirmed by the XRD patterns shown in Figure S4d. Upon further calcination and annealing, the NaNO_3_ phase disappears; however, an unidentified crystalline phase emerges near 300 °C and persists at higher temperatures. The formation of Li_3_PO_4_ and other secondary phases instead of the olivine LiMn_1–x_Ni_x_PO_4_ phase further demonstrates that the precipitation process substantially modifies the precursor stoichiometry and disrupts the compositional balance required for olivine-phase formation. Therefore, detailed characterization of the supernatant fractions was restricted to the methanol-derived systems, where precipitation is the least and the precursor composition remains sufficiently close to the intended stoichiometry to enable the formation of mesoporous olivine lithium metal phosphate phases.

**Figure 3:**
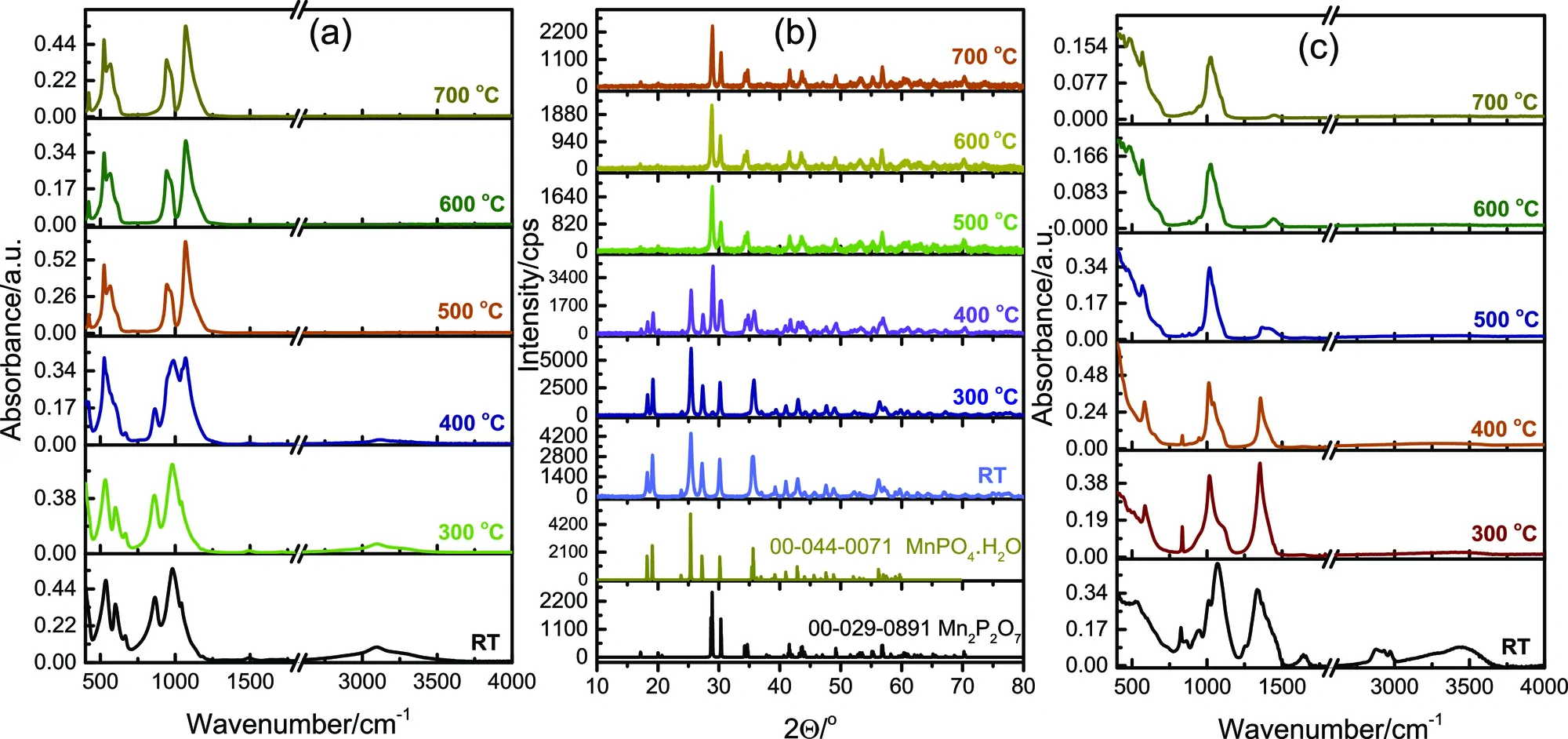
Temperature dependent ATR-FTIR spectra (a) and XRD patterns (b) of the 5% of nickel samples, obtained from the ethanol precipitate fractions, and (c) temperature dependent ATR-FTIR spectra of the same composition, derived from supernatant.

The olivine phase is successfully obtained for all compositions of the Ni­(II)–Mn­(II) systems prepared in ethanol, methanol, and even 1-butanol from their solution fractions. Figure 4a displays the XRD patterns of the calcined Ni-containing samples derived from the 1-butanol solutions. As observed throughout this study, crystalline MnPO_4_·H_2_O forms as a precipitate at RT in all Ni­(II)-containing compositions. Upon calcination, the MnPO_4_·H_2_O phase undergoes thermal transformation, producing crystalline olivine lithium mixed metal phosphate (MnNiX) together with Mn_2_P_2_O_7_ phases. In the 5 mol % Ni­(II) system, the diffraction patterns closely resemble those of o-LiMnPO_4_, particularly at temperatures above 600 °C. Increasing the Ni­(II) content results in sharper and better-resolved diffraction lines, indicating enhanced crystallinity of the olivine phase with a reduced MnPO_4_·H_2_O phase. Notably, the MnNi25 sample prepared from 1-butanol solution yields a nearly pure olivine phase. The addition of Ni­(II) significantly suppresses precipitation, suggesting that Ni­(II) improves the stability of Mn­(II) species in the precursor solution. The ATR-FTIR spectra exhibit the characteristic phosphate vibrations in the 1200–500 cm^–1^ region at all calcination temperatures. Unlike the corresponding supernatant-derived samples, no evidence of carbonate formation or ion-exchange processes (Na^+^ exchange with glass substrate) is observed in the solution-derived materials.

**Figure 4:**
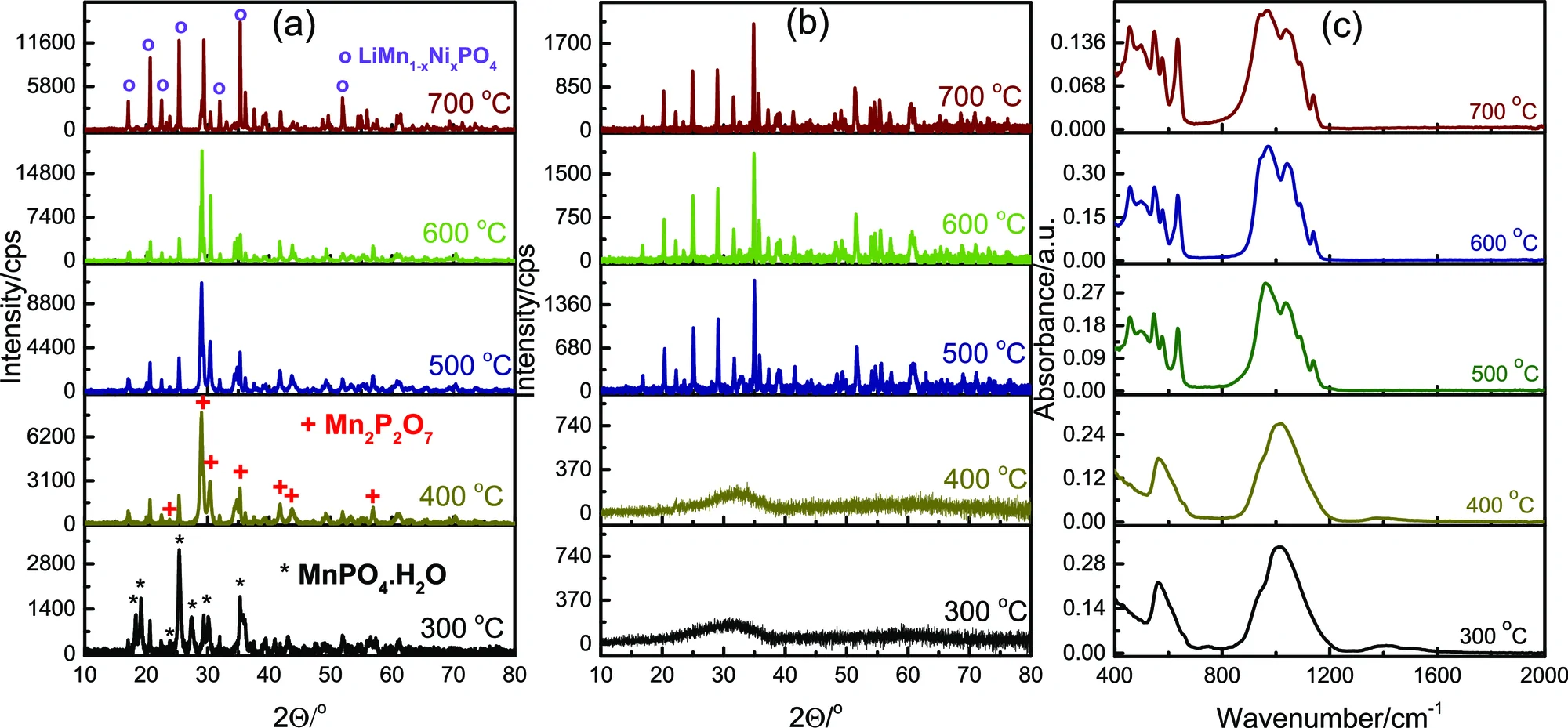
Temperature dependent XRD patterns of MnNi5-Y: (a) 1-butanol and (b) methanol solutions. (c) Temperature dependent ATR-FTIR spectra of MnNi25-Y methanol supernatant.

The gels obtained from the ethanol- and 1-butanol-derived solutions exhibit diffraction lines corresponding to crystalline MnPO_4_·H_2_O with the olivine phase, whereas no crystalline reflections are observed in the XRD pattern of the calcined gel of the methanol solution (Figure 4b). Upon calcination, all samples crystallize into the olivine LiMn_1–x_Ni_x_PO_4_ phase over 700 °C; however, the methanol-derived samples display the highest purity. Remarkably, even a 5 mol % Ni­(II) substitution is sufficient to completely suppress Mn_2_P_2_O_7_ formation in the methanol system. In contrast, the ethanol- and 1-butanol-derived samples consistently contain minor amounts of Mn_2_P_2_O_7_ at elevated temperatures. These findings strongly suggest that pyrophosphate formation originates from the thermal convrsion of precipitated MnPO_4_·H_2_O into Mn_2_P_2_O_7_ above 400 °C. Therefore, stabilization of Mn­(II) in the precursor solution and concomitant suppression of MnPO_4_·H_2_O precipitation are key prerequisites for the synthesis of phase-pure olivine LiMn_1–x_Ni_x_PO_4_ materials.

Owing to the limited amount of precipitate formed in methanol, the supernatant and solution fractions generate essentially identical products (Figures S5 and S6). Figure S5 shows the XRD patterns of the MnNiX-Y samples prepared from methanol and ethanol solutions and calcined at different temperatures. The methanol-derived materials consist solely of crystalline olivine LiMn_1–x_Ni_x_PO_4_, whereas the ethanol-derived materials are mesoporous and comprise a dominant olivine phase together with small amounts of secondary phases. Figure S6 presents the XRD patterns and ATR-FTIR spectra of the calcined products obtained from the methanol-derived supernatants. The material remains essentially amorphous up to 500 °C and subsequently crystallizes into a phase-pure olivine lithium manganese–nickel phosphate (o-Li-Mn_1–x_Ni_x_PO_4_) at higher temperatures. The crystallization process is also reflected in the ATR-FTIR spectra, where the phosphate vibrational bands become sharper and better resolved with increasing calcination temperature (Figure 4c), indicating the progressive development of long-range structural order. Figure S6d compares the XRD patterns of samples containing different amounts of Ni­(II). A systematic shift of the diffraction lines toward higher 2θ values is observed with increasing Ni­(II) content. This behavior is consistent with the substitution of Mn­(II) by the smaller Ni­(II) ions within the olivine lattice, resulting in a gradual contraction of the unit cell. The absence of additional diffraction lines and the continuous line shift strongly suggest that Ni­(II) is homogeneously incorporated into the LiMn_1–x_Ni_x_PO_4_ structure, leading to the formation of a solid-solution phase over the investigated compositional range.

Morphologies of the precipitates were investigated by collecting SEM images of representative MnNi25-Y (ethanol), MnNi5-Y (methanol), MnNi5-Y (ethanol), and MnNi5-Y (1-butanol) samples annealed at 300 and 700 °C (Figure 5). The particles remain largely unchanged upon annealing up to 700 °C. At 700 °C, the particles begin to sinter and become partially fused to one another, indicating the onset of pore collapse and densification. Nevertheless, unlike the LiMnPO_4_ system, the particles do not coalesce into continuous film-like structures.^34^ Instead, they retain their particulate nature and exhibit a grainy surface texture, suggesting that the presence of Ni­(II) enhances the thermal stability of the structure and improves its resistance to morphological degradation during heat treatment.

**Figure 5:**
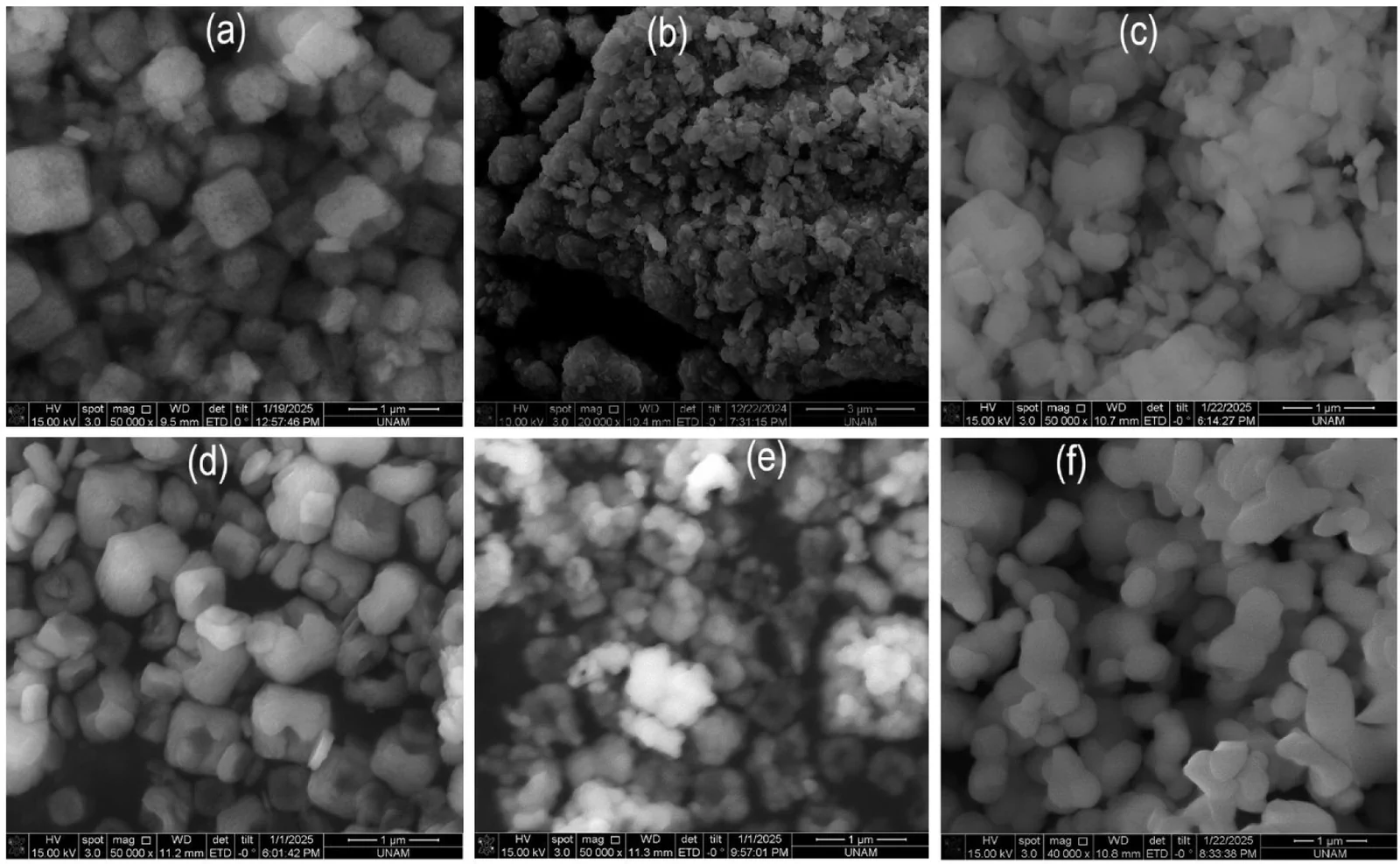
SEM images of the precipitates from (a) MnNi25-300 (ethanol), (b) MnNi5-300 (methanol), (c) MnNi5-300 (ethanol), (d) MnNi5-300 (1-butanol), (e) MnNi5-700 (1-butanol), and (f) MnNi5-700 (ethanol). Scale bars are 1 μm in all images.

The higher crystallinity of the 1-butanol-derived samples, as inferred from the XRD patterns, is also reflected in their morphology (Figure 5d). The choice of solvent plays a critical role in controlling both the crystallization behavior and the final morphology of the products. Among the solvents investigated, 1-butanol appears to be particularly advantageous for producing larger and better-developed MnPO_4_·H_2_O crystals. This behavior is likely related to its lower polarity and slower evaporation rate, which provide a more controlled environment for nucleation and crystal growth, ultimately promoting the formation of highly crystalline faceted particles.

Figure 6 presents the N_2_ adsorption–desorption isotherms and pore-size distribution plots of the calcined MnNi5 precipitates obtained from ethanol and 1-butanol, together with the MnNi25-300 precipitate and solution-derived samples prepared in 1-butanol. All four samples exhibit type-IV adsorption isotherms, characteristic of mesoporous materials.^49^ However, significant differences are observed in their surface areas and pore structures. The MnNi5-300 precipitate obtained from ethanol possesses a relatively low surface area of only 4 m^2^ g^–1^ and an average pore diameter of approximately 5.1 nm. In contrast, the corresponding 1-butanol-derived precipitate exhibits a higher surface area (12 m^2^ g^–1^) together with a larger average pore size of 5.8 nm. These results indicate that the ethanol-derived precipitate develops a more compact and less porous structure, even at relatively low calcination temperatures. Increasing the Ni­(II) content further enhances the textural properties of the material. The MnNi25-300 precipitate exhibits a significantly higher surface area of 29 m^2^ g^–1^ and a narrower pore-size distribution centered at approximately 3.3 nm, indicating the formation of a more uniform mesoporous network. Increasing surface area with increasing Ni­(II) media aligns well with the amount of large MnPO_4_·H_2_O crystallites that decreases with increasing Ni­(II) amount in the initial solutions. The MnNi25-300 sample largely preserves its structural integrity and particle morphology, whereas the MnNi5-300 sample develops a more irregular and less well-defined microstructure. These observations further demonstrate that both solvent selection and Ni content play critical roles in controlling the textural evolution, crystallization behavior, and thermal stability of the resulting olivine materials.

**Figure 6:**
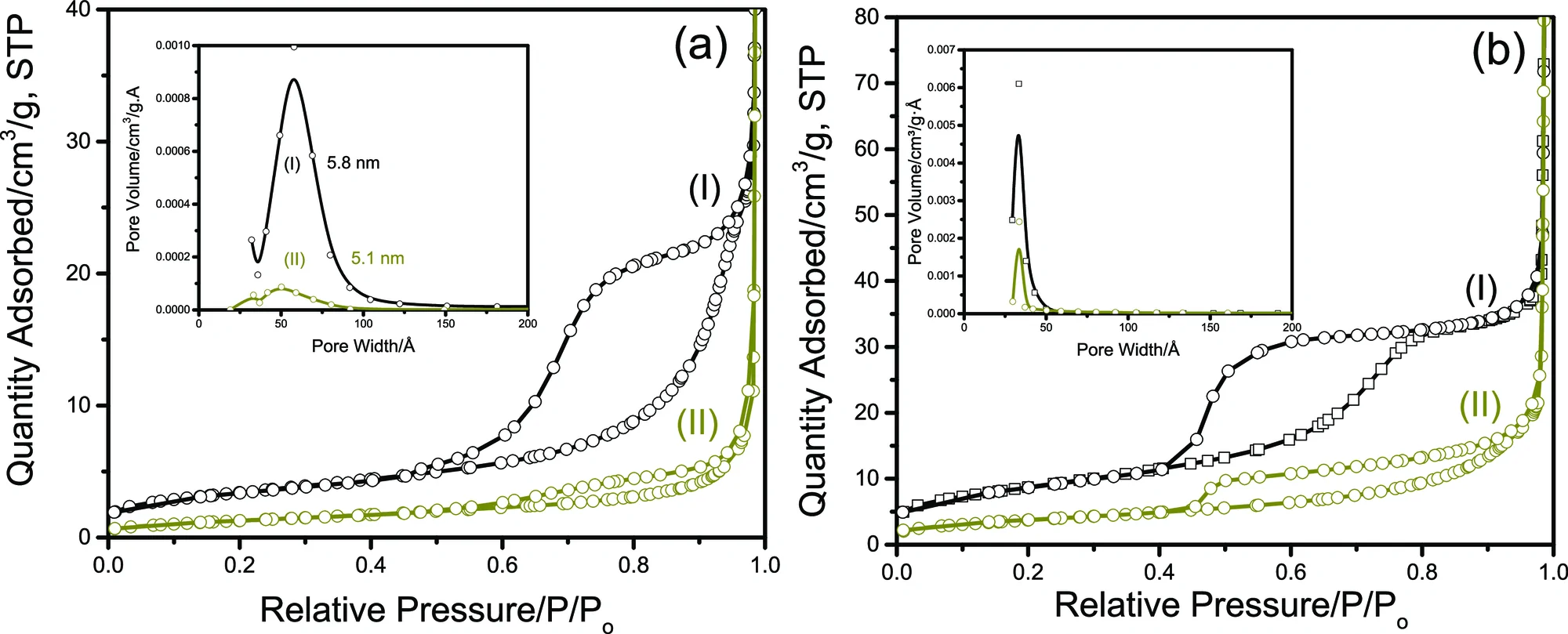
N_2_ adsorption–desorption isotherms (insets indicate the pore size distribution plots) of (a) MnNi5-300 (from 1-butanol (I) and ethanol (II) precipitates) and (b) MnNi25-300 (1-butanol) precipitate (I) and solution (II).

SEM images of the products, obtained from the methanol-derived solutions, are compared in Figure 7. Because precipitation is minimal in methanol, the Ni­(II)/Mn­(II) ratio remains much closer to the initial precursor composition than in the ethanol- and 1-butanol-based systems. Consequently, the final products more accurately reflect the intended stoichiometry of the starting solutions. The images illustrate the effect of increasing Ni­(II) content on the morphology of the materials synthesized from methanol solutions (Figure 7). The most striking difference compared with the ethanol- and 1-butanol-derived samples is the predominantly spherical morphology of the particles. Relative to the particles obtained from the pure Mn­(II) system, the mixed-metal particles are generally larger, exhibit rougher surfaces, and show a greater tendency to aggregate. The MnNi5 sample consists of relatively large and highly spherical particles, whereas increasing the Ni­(II) content leads to a gradual reduction in particle size. The effect of calcination temperature on particle morphology was also investigated for the MnNi25 sample. Upon increasing the calcination temperature from 300 to 700 °C, progressive particle sintering is observed, accompanied by crystallization and partial pore collapse.

**Figure 7:**
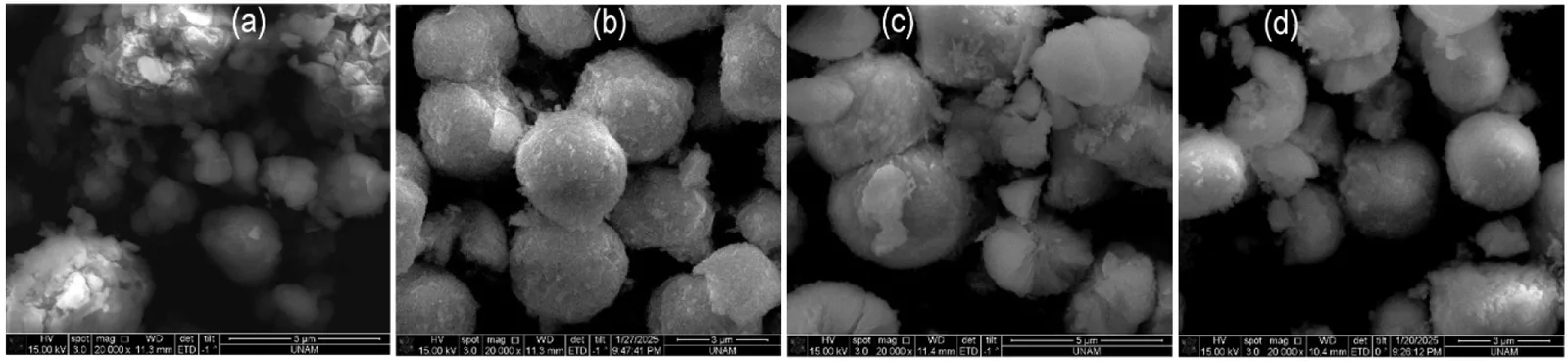
SEM images of the samples calcined at 300 °C, from methanol solutions of MnNiX, X is (a) 0 (scale bar is 5 μm), (b) 0.05 (scale bar is 3 μm), (c) 0.1 (scale bar is 5 μm), and (d) 0.25 (scale bar is 3 μm).

The samples obtained from the methanol-derived solutions and supernatants were further investigated for their textural properties using N_2_ adsorption–desorption measurements, along with BET surface-area and BJH pore-size analyses. The surface areas and pore volumes of the methanol-derived samples are summarized in Table 1. The results indicate that the Ni­(II) content has only a minor influence on the surface area and pore structure of both the solution-derived and supernatant-derived materials. Regardless of composition, the samples exhibit comparable textural characteristics, suggesting that Ni incorporation does not significantly affect the mesostructural evolution in the methanol system. Nevertheless, the supernatant-derived samples consistently exhibit slightly higher surface areas than their solution-derived counterparts. The reduced precipitation in the methanol system also helps preserve the homogeneity of the precursor medium, leading to more effective pore development during calcination.

**Table 1:** BET Surface Area and BJH Pore Volume Values of the Methanol Solutions and Supernatants of the MnNiX-300 Samples

Samples	Fractions	Surface Area (m^2^/g)	Pore Volume (cm^3^/g)
MnNi5-300	Supernatant	26	0.26
MnNi5-300	Solution	18	0.26
MnNi10-300	Supernatant	20	0.30
MnNi10-300	Solution	17	0.25
MnNi25-300	Supernatant	24	0.24
MnNi25-300	Solution	23	0.27

### Mesoporous LiMn_1–x_Co_x_PO_4_ (x = 0.05, 0.10, and 0.25)

A brown precipitate also forms in all Co­(II)-containing solutions regardless of the solvent used. Similar to the Ni­(II)-containing systems, crystalline MnPO_4_·H_2_O precipitates at RT and subsequently transforms into Mn_2_P_2_O_7_ upon calcination above 400 °C for all investigated Co­(II) compositions (Figure S7). Among the solvents examined, methanol yields the smallest amount of precipitate. Therefore, both the solution and supernatant fractions were characterized for the methanol system, whereas only the precipitates and solution-derived materials were investigated for the ethanol- and 1-butanol-based systems.

The ethanol-, methanol-, and 1-butanol-derived LMnCo25P samples were further characterized after calcination (MnCo25-Y, Y is the calcination temperature). The XRD patterns and ATR-FTIR spectra exhibit trends similar to those observed for the corresponding Ni-containing systems. The olivine lithium metal phosphate phase forms in all MnCoX-Y compositions (X = 5, 10, and 25 mol % Co) regardless of the solvent type. Incorporation of Co­(II) results in only minor shifts in the diffraction lines, indicating that Co­(II) is homogeneously incorporated into the olivine lattice and forms a solid-solution phase rather than segregating into separate crystalline domains.

Because precipitation is minimal in methanol, both the solution- and supernatant-derived samples were investigated by collecting their XRD patterns and ATR-FTIR spectra at various calcination temperatures. Figure 8 presents the XRD patterns of these samples. Both materials crystallize at approximately 300 °C, forming the olivine phase. Selected regions of the XRD patterns are also shown for comparison with the pure LiMnPO_4_ and LiCoPO_4_ end members (Figure 8c and d). In both the solution- and supernatant-derived samples, the diffraction lines progressively shift toward higher diffraction angles with increasing Co­(II) incorporation, indicating the substitution of larger Mn^2+^ ions by smaller Co^2+^ ions within the LiMnPO_4_ lattice (Figures 8 and S8). This conclusion is further supported by the calculated unit cell parameters and unit cell volumes (Table S2). The unit cell volumes of the two end members, LiMnPO_4_ and LiCoPO_4_, are 298.6 and 279.6 Å^3^, respectively, whereas that of LiMn_0.75_Co_0.25_PO_4_ is 292.5 Å^3^. Assuming a linear dependence of unit cell volume on composition (Vegard’s law), this value corresponds to approximately 32% Co incorporation, which is in good agreement with the nominal composition. This trend is also evident from the XRD peak shifts, which are slightly more pronounced for the supernatant-derived samples. The larger shift can be attributed to the higher Co^2+^/Mn^2+^ ratio in the supernatant, resulting from the preferential precipitation of Mn as MnPO_4_·H_2_O during the synthesis. Consequently, the supernatant becomes enriched in Co^2+^, leading to a higher degree of Co incorporation into the olivine lattice. Furthermore, the mesoporous material derived from the methanol supernatant exhibits phase behavior nearly identical to that of the corresponding solution-derived sample, demonstrating that the limited extent of precipitation in methanol does not significantly alter either the crystallization pathway or the final crystal structure. This finding further indicates that the precursor composition in methanol remains sufficiently close to that of the initial solution despite partial MnPO_4_·H_2_O precipitation.

**Figure 8:**
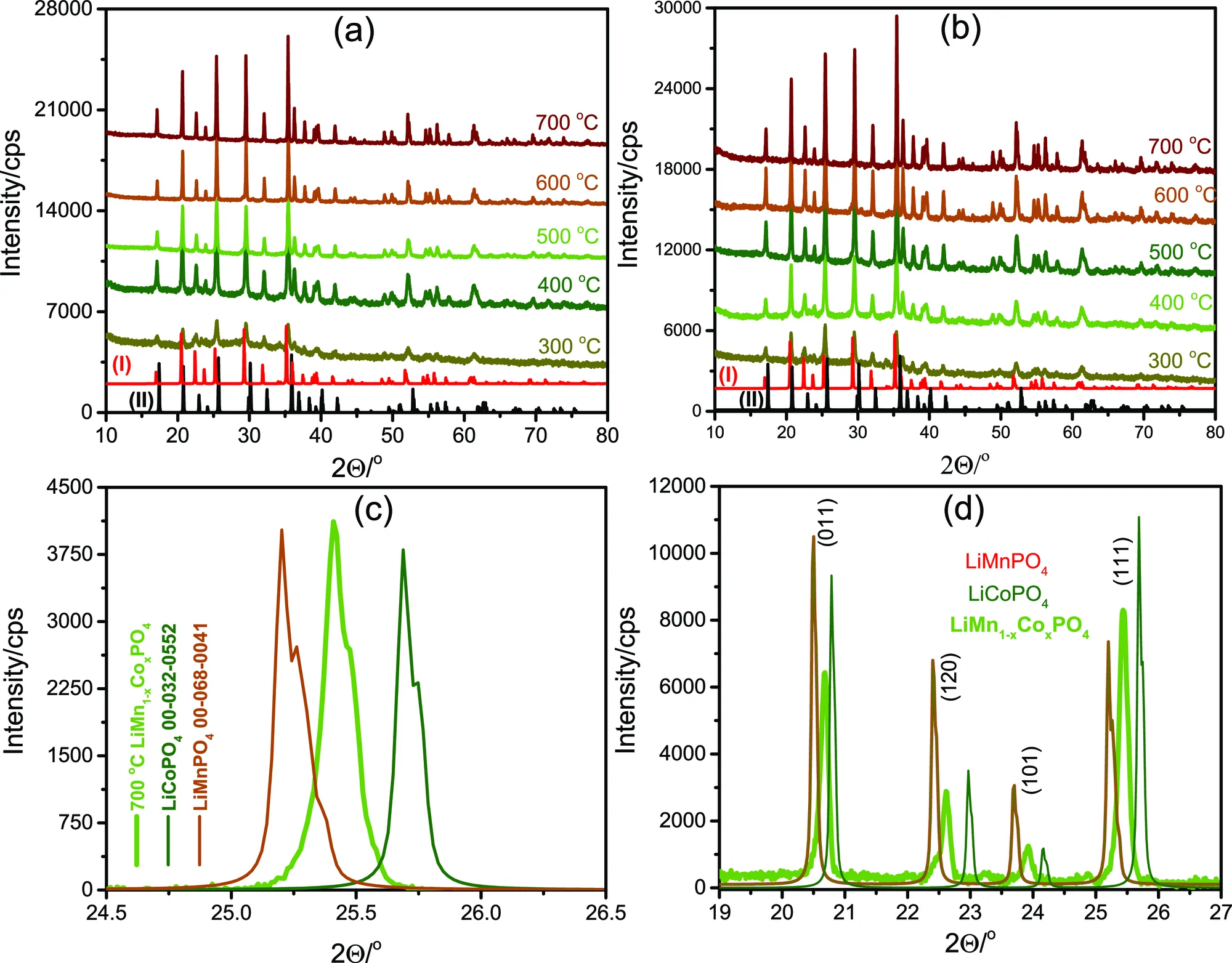
Temperature dependent XRD pattern of MnCo25-Y derived from methanol (a) solution and (b) supernatant ((I) LiMnPO_4_ (00-068-0041) and (II) LiCoPO_4_ (00-032-0552), and XRD pattern from ICDD cards of LiMnPO_4_ (00-068-0041), LiCoPO_4_ (00-032-0552), and LiMn_0.75_Co_0.25_PO_4_-700 derived from (c) solution, (111) line) and (d) supernatant fraction.

The ATR-FTIR spectra shown in Figure S8d are also consistent with those obtained for the MnNiX-Y solution-derived samples. The nitrate- and water-related bands diminish upon calcination at 300 °C, and no residual nitrate or carbonate species are detected in the final products. The olivine framework is already established at 300 °C and remains structurally stable upon further heat treatment. Accordingly, no significant changes are observed in the phosphate vibrational region, although the bands become sharper and better resolved with increasing calcination temperature. This spectral evolution reflects the progressive increase in crystallinity and structural ordering of the olivine framework during thermal treatment.

SEM images of the MnCo25-Y samples obtained from the different solvent systems were collected to investigate the influence of solvent on morphological evolution during calcination. Since, methanol solution and supernatant produce pure olivine phases, the methanol-derived samples are further investigated by collecting their SEM images. At 300 °C, the methanol-derived samples consist of relatively uniform and spherical particles, in contrast to the materials prepared from ethanol and 1-butanol. Upon further calcination, the methanol-derived solution and supernatant samples develop porous structures that are partially retained even at elevated temperatures (Figure 9). In contrast, the ethanol-derived samples exhibit nonuniform particles. The influence of precipitation is even more pronounced in the 1-butanol system, where larger and highly faceted particles are observed even at relatively low calcination temperatures (Figure S9). These particles display well-defined cubic or polyhedral morphologies due to MnPO_4_·H_2_O crystals. However, similar to the ethanol-derived samples, extensive sintering and pore collapse occur at 700 °C, resulting in dense aggregated structures.

**Figure 9:**
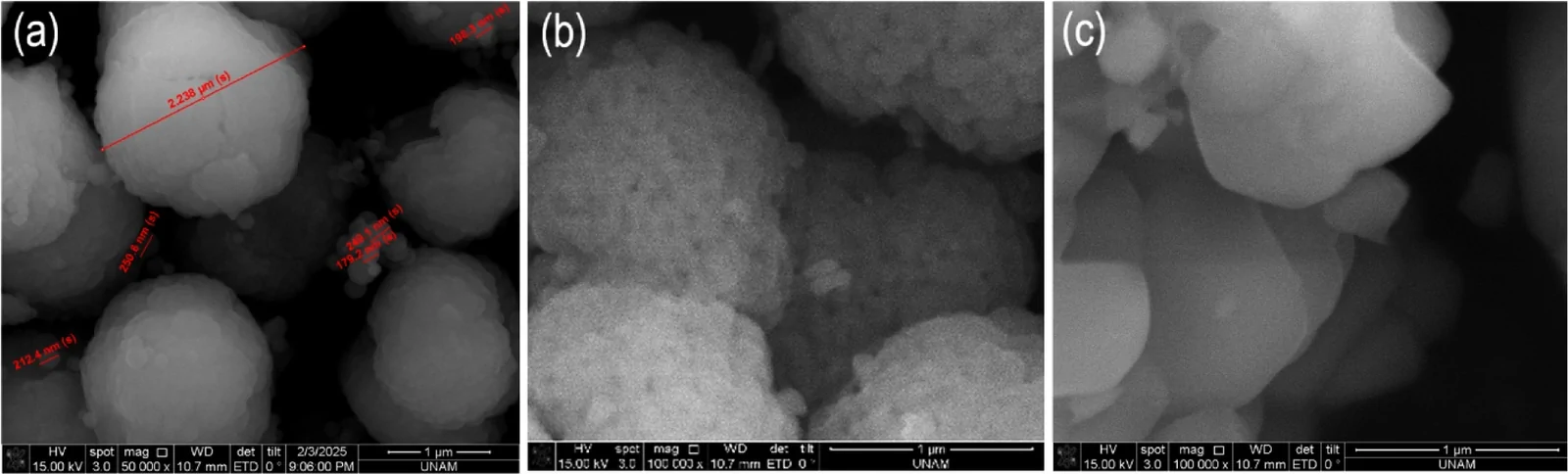
SEM images of the methanol solution-derived MnCo25-Y samples, Y is (a) 300, (b) 500, and (c) 700 (scale bars are 1 μm).

A comparison of the two binary-metal systems reveals distinct solvent-dependent morphological trends. In both the MnNi25-Y and MnCo25-Y systems, methanol favors the formation of smaller, more spherical, and more uniform particles, whereas 1-butanol promotes the growth of larger faceted grains. Furthermore, the MnNi25-Y samples generally consist of finer and less aggregated particles regardless of the solvent used, particularly at lower calcination temperatures. In contrast, the MnCo25-Y samples exhibit more pronounced crystallinity and larger crystal domains. This behavior is consistent with the slower nucleation and faster crystal-growth kinetics reported for Co-containing systems relative to their Ni-containing counterparts, leading to the formation of larger and more crystalline particles.

Furthermore, the methanol-derived MnMX-Y materials and their corresponding precipitates were investigated by XPS to gain insight into their surface compositions and oxidation states. In particular, the LiMn_0.75_Ni_0.25_PO_4_ and LiMn_0.75_Co_0.25_PO_4_ samples obtained from the methanol supernatants were analyzed prior to electrochemical measurements (Figure S10). The XPS results indicate that the transition-metal species are predominantly present in their +2 oxidation states. This assignment is further supported by the Mn 3s spectra, which are consistent with Mn­(II) as the dominant manganese species.^50^ The Mn 2p spectra exhibit a weak shoulder near 642 eV, suggesting the presence of a small amount of Mn­(III) species on the surface. However, the overall spectral features indicate that Mn­(II) remains the predominant oxidation state. In addition, the O 1s and P 2p spectra are consistent with phosphate-based materials and closely resemble the compositions expected from the bulk phases. These observations suggest that the surface composition accurately reflects the bulk composition of the materials, with no significant surface segregation or enrichment of secondary phases. Consequently, the XPS results confirm that the methanol-derived LiMn_0.75_Ni_0.25_PO_4_ and LiMn_0.75_Co_0.25_PO_4_ samples consist primarily of homogeneous mixed-metal phosphate phases containing transition metals in their divalent oxidation states prior to electrochemical tests.

### Electrochemical Behaviors of the Mesoporous LiMn_1–x_M_x_PO_4_ (M = Ni or Co) Electrodes

The precursor solutions of the binary-metal systems were coated onto FTO and graphite substrates to fabricate the LiMn_1–x_M_x_PO_4_ (M = Ni or Co and x = 0.05, 0.10, and 0.25) electrodes. FTO-coated electrodes were prepared by spin-coating at 5000 rpm (denoted as FTO-MnMX-Y, X is secondary metal percent, Y is calcination/annealing temperature in Celsius), whereas graphite-rod electrodes (GR-MnMX-Y) were fabricated by dip-coating from solutions diluted 5-fold and immersing the substrate for 5 s with a pulling-speed of 1.45 mm/s. The coated substrates were subsequently calcined at temperatures between 300 and 500 °C. Electrochemical measurements were carried out in 1 M KOH using a conventional three-electrode configuration.

To evaluate their electrochemical behaviors, 50 consecutives cyclic voltammograms were collected within a potential window of −0.5 to 2.0 V (vs NHE) with a scan-speed of 50 mV/s for the ethanol-derived (namely FTO-MnNi5 and FTO-MnCo5) electrodes (Figure S11). The incorporation of 5 mol % Ni­(II) significantly improves the stability of the FTO-MnNi5-300 electrode, enabling the collection of 50 consecutive cycles without severe degradation. In contrast, the FTO-MnCo5-300 electrode containing 5 mol % Co­(II) exhibits insufficient stability on the FTO substrate and fails to withstand prolonged cycling under the same conditions. Furthermore, increasing the calcination temperature reduces the stability of the FTO-MnNi5-Y electrodes, particularly in the OER potentials, between 0.8 and 2.0 V. The CVs display the characteristic redox features associated with the electrochemical transformation of Ni­(OH)_2_/NiOOH and Co­(OH)_2_/CoOOH species formed under alkaline conditions. However, the measured peak currents remain relatively low, typically in the range of 2–3 mA cm^–2^. These results indicate that although the incorporation of Ni­(II) improves electrode stability relative to Co­(II), the FTO-supported electrodes possess limited electrocatalytic activity and durability under OER operating conditions, particularly after high-temperature calcination.

Because significant stability issues were observed for the FTO-supported electrodes, all subsequent electrochemical investigations were carried out using graphite rod (GR) electrodes. Figure 10 presents a representative set of 50 consecutive cyclic voltammograms recorded for the ethanol-derived GR-MnNi10-Y (Y = 300 and 400) electrodes in 1 M KOH solution. The CVs clearly demonstrate that the electrodes progressively reconstruct during cycling, evidenced by the continuous growth of the Ni^2+^/Ni^3+^ redox feature associated with the conversion of lithium metal phosphate to mixed-metal hydroxides. This reconstruction occurs for all electrodes irrespective of the calcination temperature; however, the transformation rate decreases with increasing annealing temperature, suggesting that the more crystalline materials have more resistance to alkaline-induced reconstruction. The other compositions display analogous behavior.

**Figure 10:**
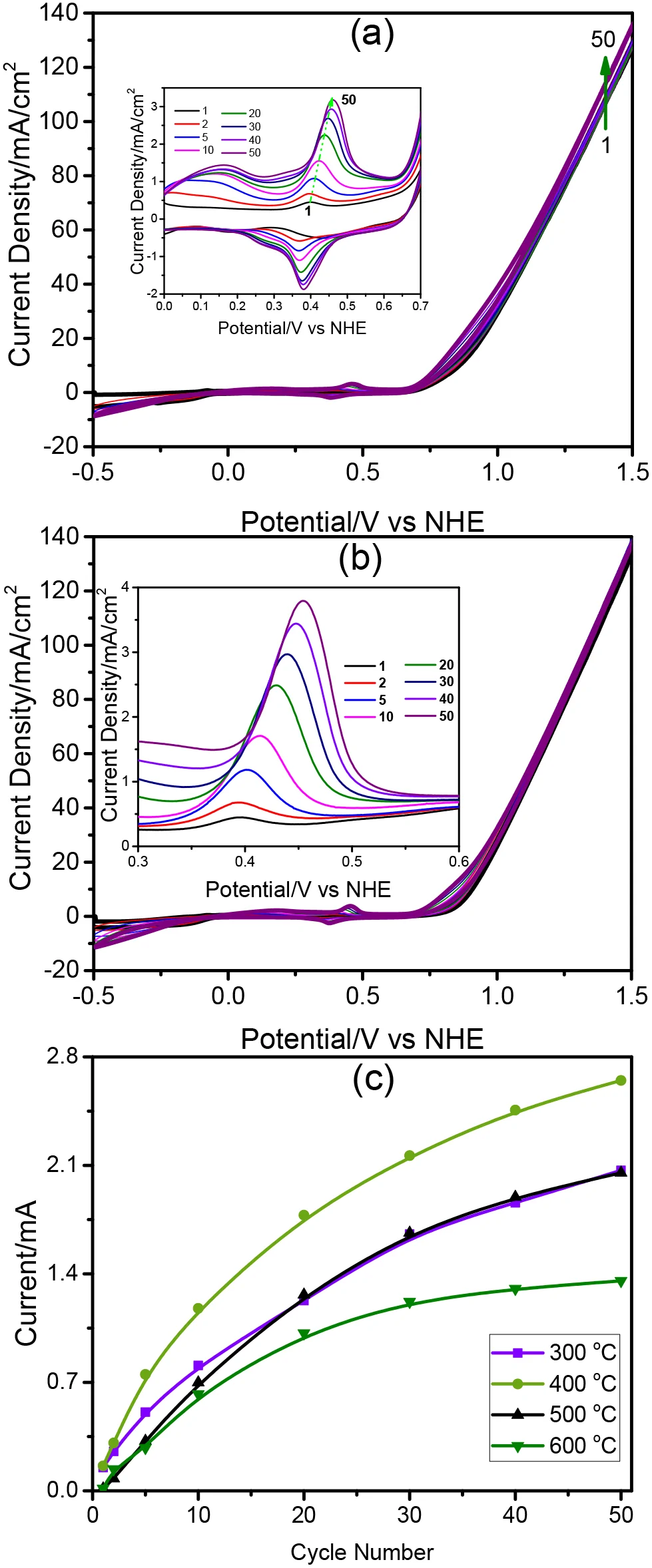
Selected CVs from a set of 50 consecutive CV cycles (scan rate of 50 mV/s) of the ethanol-derived (a) GR-MnNi10-300, (b) GR-MnNi10-400, and (c) plot of oxidation peak current versus CV cycle number (time) during CV measurements.

Similar to the behavior observed for the pure Mn-based electrodes,^36^ partial electrode decomposition occurs during the OER on the mixed-metal phosphate electrodes (Figure S12). Under strongly alkaline and oxidative conditions, Mn­(II) is progressively oxidized to higher oxidation states and ultimately forms soluble permanganate (MnO_4_^–^), which diffuses into the electrolyte. This process is readily observed by the appearance of a pink-to-purple coloration in the electrolyte solution during electrochemical cycling (Figure S12). The incorporation of Ni­(II), however, substantially suppresses this degradation pathway. In most cases, the release of permanganate is primarily observed during the first 3–5 cycles, after which the electrodes, particularly those calcined at lower temperatures, exhibit significantly improved stability. Notably, the electrode calcined at 500 °C does not produce any visible coloration associated with Mn­(VII) formation, indicating that higher calcination temperatures enhance structural stability and effectively inhibit manganese dissolution. These observations demonstrate that calcination temperature is a critical parameter for obtaining stable GR-LiMn_1–x_Ni_x_PO_4_ electrodes under OER conditions.

The stability and catalytic performance of the electrodes were further evaluated in the water-oxidation potentials. Stable electrodes can be obtained for all compositions of the Ni-containing binary-metal system. Among them, the GR-MnNi25-Y electrodes exhibit the highest current densities, indicating that increased Ni content enhances OER activity (Figure S13). Furthermore, as the Ni­(II) concentration increases, the characteristic Ni­(OH)_2_/NiOOH redox couple becomes increasingly pronounced (Figure S13). The anodic peak near 0.5 V corresponds to the oxidation of Ni­(OH)_2_ to NiOOH, while the cathodic peak near 0.3 V is associated with the reverse reduction process (Figure 10a). The increasing current density of these redox features with increasing Ni content suggests that a larger fraction of electrochemically active Ni hydroxide species is formed during cycling, which likely contributes to the improved catalytic performance and stability of the high-Ni electrodes. Figure 10c shows plots of the oxidation peak current as a function of cycle number, illustrating the evolution of the Ni^2+^/Ni^3+^ redox peak associated with the transformation of the phosphate phase into mixed-metal hydroxides.

The methanol- and 1-butanol-derived LiMn_1–x_Ni_x_PO_4_ electrodes were also prepared following the same procedure and evaluated under identical electrochemical conditions. Representative sets of 50 consecutive CVs recorded for the GR-MnNiX (X = 0.05, 0.10, and 0.25) electrodes are presented in Figures S14 and S15, respectively. The results clearly demonstrate that both the solvent used during synthesis and the Ni­(II) content significantly influence the OER activity and stability of the electrodes.

The methanol-derived GR electrodes exhibit excellent electrochemical performance and stability. For all Ni compositions and calcination temperatures investigated, the current density approaches approximately 120 mA cm^–2^ at 1.5 V (vs NHE) during cycling while maintaining a stable electrochemical response over 50 cycles. Importantly, the current density obtained at 1.6 V (vs RHE) exceeds by over 70% those of similar electrodes reported in the literature.^51^ In contrast, the ethanol-derived electrodes also display good cycling stability across all compositions and calcination temperatures but, exhibit the lowest catalytic activity among the three solvent systems. The highest OER performance is obtained from the 1-butanol-derived electrodes. In these samples, the current density increases progressively during repeated cycling for nearly all Ni compositions, suggesting an electrochemical activation process occurring at the electrode surface. This behavior is likely associated with the gradual formation of catalytically active hydroxide/oxyhydroxide species during cycling. In addition, the dissolution of Mn through the Mn­(VI)/Mn­(VII) pathway enriches the electrode surface in Ni-containing species, thereby increasing the relative abundance of electrochemically active Ni­(OH)_2_/NiOOH sites, thus enhancing OER activity.^28^

The effect of calcination temperature is particularly pronounced for the GR-MnNi25 electrodes. As the annealing temperature increases, the current density measured at 1.5 V (vs NHE) decreases from approximately 100 mA cm^–2^ to 80 mA cm^–2^. This reduction in activity may be attributed to enhanced crystallinity and reduced surface area of the electrode material at higher calcination temperatures, which likely limits the formation of the electrochemically active Ni­(OH)_2_/NiOOH surface layer during cycling. Consequently, although higher calcination temperatures improve structural stability, they may also reduce the density of accessible active sites responsible for oxygen evolution.

Overall, the electrochemical results indicate that both solvent selection and thermal treatment strongly influence the balance between structural stability and catalytic activity. Among the investigated solvents, 1-butanol produces the most active electrodes, whereas methanol yields the most stable performance with consistently high current densities over prolonged cycling.

The transformation of the MnMX electrodes (M = Ni or Co) into mixed-metal hydroxides, (Mn,M)­(OH)_2_, can be directly monitored by wide-angle XRD and ATR-FTIR spectroscopy (Figures S16 and S17). The ATR-FTIR spectra reveal that the transformation begins immediately upon immersion of the electrodes in 1 M KOH. In the case of the MnCo25 electrode, hydroxide-related vibrations emerge within 15 min of exposure to the alkaline electrolyte, as evidenced by the appearance of the sharp O–H stretching band at 3620 cm^–1^. Simultaneously, the phosphate vibrational bands progressively decrease in intensity with increasing immersion time. These observations demonstrate that the conversion of the phosphate framework into hydroxide phases is primarily a chemically driven process induced by the alkaline medium rather than an electrochemically triggered transformation.^34,35^

It should be noted that the XRD and ATR-FTIR experiments were performed by immersing 100 mg of MnMX-300 powder in 10 mL of 1 M KOH solution. In contrast, the electrochemical measurements were conducted in 20 mL of 1 M KOH using only 0.05–0.10 mg of active material coated on the electrode surface. Therefore, the electrolyte-to-solid ratio during electrochemical measurements is several orders of magnitude higher than that employed in the *ex-situ* transformation studies. Consequently, the hydroxide reconstruction of the electrodes is expected to proceed much more rapidly under electrochemical conditions and is likely completed during the initial CV cycles, as observed in the plots in Figure 10c.

A similar behavior is observed for the GR-MnNi25-Y electrode. Although a distinct hydroxide band near 3620 cm^–1^ is not readily resolved, a progressive increase in intensity within the O–H stretching region is evident as the immersion time increases in 1 M KOH. At the same time, the phosphate vibrational bands in the 1250–750 cm^–1^ region continuously lose intensity (Figure S16), indicating the gradual consumption of the phosphate phase and formation of hydroxide species.

The XRD patterns further support the conclusions drawn from the ATR-FTIR measurements. For both the MnCo25-300 and MnNi25-300 samples, structural changes are observed immediately after contact with the alkaline electrolyte, demonstrating that the transformation to mixed (Mn,M)­(OH)_2_ phase begins spontaneously upon immersion in 1 M KOH and completes by fully converting MnMX to (Mn,M)­(OH)_2_. However, in the presence of a relatively large amount of lithium metal phosphate (100 mg in 10 mL of 1 M KOH), the conversion is not instantaneous and remains incomplete even after 3 h of exposure (Figure S17). The coexistence of phosphate and hydroxide diffraction features during this period indicates that the transformation proceeds through a gradual dissolution–reprecipitation process, in which the original phosphate framework is progressively replaced by layered mixed-metal hydroxide domains.

It should be noted that the extent of the transformation is strongly governed by the [PO_4_^–^]/[OH^–^] ratio. In the *ex-situ* experiments, the large amount of phosphate material results in a relatively high phosphate concentration, thereby slowing the conversion process. In contrast, the electrochemical measurements employ only 0.05–0.10 mg of active material in 20 mL of 1 M KOH, yielding an extremely low [PO_4_^–^]/[OH^–^] ratio. Under these conditions, complete hydroxide reconstruction is expected to occur rapidly and is likely to be completed during the initial CV cycles.^28^ These results clearly demonstrate that the electrochemically active phases under alkaline conditions are not the original lithium manganese–transition-metal phosphates but rather the mixed (Mn,M)­(OH)_2_ phases formed in situ upon exposure to the electrolyte.

The MnCoX-Y electrodes were also prepared from ethanol-, methanol-, and 1-butanol-derived precursor solutions to investigate the influence of Co­(II) incorporation on the stability and electrocatalytic performance of the LiMnPO_4_ electrodes. Similar to the observations made for the Ni-containing systems, the FTO-MnCoX-Y electrodes exhibited poor stability under alkaline OER conditions (Figure S18), whereas the graphite rod-supported electrodes showed significantly improved durability. Therefore, the electrochemical properties of the GR-MnCoX-Y electrodes synthesized from the different solvent systems were investigated in detail.

Figure S19 presents a representative set of 50 consecutive CVs recorded for the ethanol-derived GR-MnCoX-Y (X = 5, 10, and 25 mol %) electrodes. The results demonstrate that increasing the Co­(II) content substantially improves the stability of the electrodes, regardless of the calcination temperature. Among all compositions examined, the GR-MnCo25 electrodes exhibit the highest stability and achieve the largest current densities, indicating superior OER activity.

The GR-MnCo5 electrode calcined at 300 °C undergoes significant degradation during the first 20 CV cycles. When calcined at 400 °C, the current density initially increases and reaches a maximum around the 30th cycle. However, further cycling leads to a gradual decline in activity, as indicated by the arrows in Figure S19. This behavior may reflect a surface passivation process occurring after prolonged cycling. During the initial cycles, Mn species are progressively oxidized and removed through the Mn­(VI)/Mn­(VII) dissolution pathway, enriching the electrode surface in Co-containing species.^28,43^ Once this restructuring process is completed, the remaining surface may become less active, resulting in a gradual decrease in current density.

In contrast, increasing the Co content to 10 mol % produces significantly more stable electrodes. Furthermore, calcination at 500 °C appears to activate the GR-MnCo10-500 electrode, resulting in a robust and stable structure capable of sustaining current densities approaching 100 mA cm^–2^ at 1.5 V (vs NHE) during cycling (Figure S19). These observations indicate that both Co incorporation and thermal treatment contribute to the stabilization of the electrode surface under OER conditions.

The electrochemical redox behavior of the electrodes also evolves with Co content. All compositions display distinct redox features in the low-potential region between approximately 0 and 0.2 V, and these signals become increasingly pronounced as the Co concentration increases. However, unlike the GR-MnCo5 and GR-MnCo10 electrodes, the GR-MnCo25 electrode exhibits no clearly discernible reduction peak. This behavior may indicate that the surface oxidation process becomes largely irreversible under the applied conditions, or alternatively, that the high OER current masks the relatively weak cathodic features. Regardless of the exact origin, the absence of a well-defined reduction peak coincides with enhanced catalytic performance and stability. Overall, the ethanol-derived GR-MnCo25 electrodes outperform the lower-Co compositions in both stability and electrocatalytic activity, even after calcination at 500 °C. These results demonstrate that increasing Co content promotes the formation of a more robust and electrochemically active surface, leading to improved performance during alkaline oxygen evolution.

Similar electrochemical experiments were performed using methanol- and 1-butanol-derived GR-MnCoX electrodes calcined at various temperatures. The corresponding CVs are presented in Figures 11 and S20. The methanol-derived GR-MnCoX-Y electrodes display the highest catalytic activity, achieving current densities approaching 165 mA cm^–2^ at 1.5 V (vs NHE). During cycling, however, the redox peaks attributed to the Co^2+^/Co^3+^ transformation gradually diminish and eventually disappear. The loss of these redox features may indicate partial delamination or segregation of the active coating from the electrode substrate during extended cycling. Alternatively, extensive surface reconstruction under alkaline conditions may obscure the original cobalt redox behavior. Despite this apparent loss of redox activity, the electrodes maintain high anodic current densities throughout the OER region (Figure S20).

**Figure 11:**
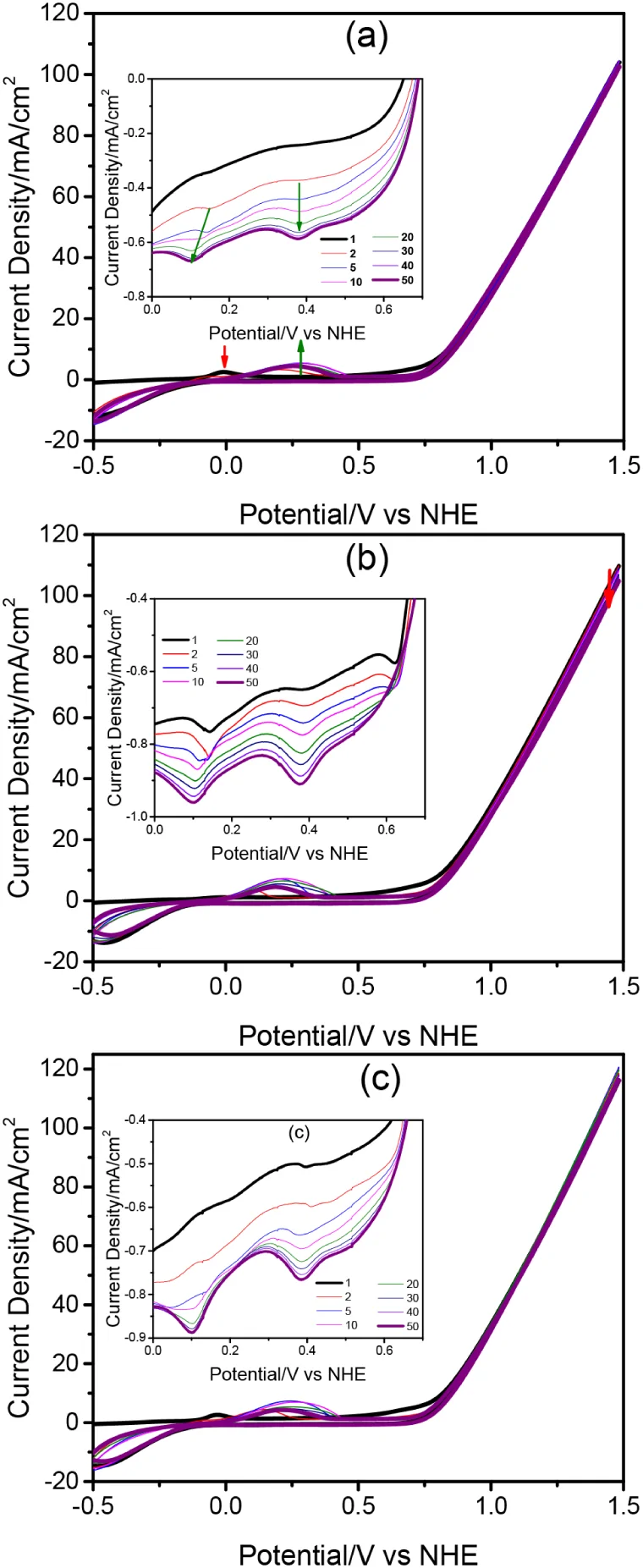
Selected CVs (1st, 2nd, 5th, 10th, 20th, 30th, 40th, and 50th) from 50 CV cycles in 1 M KOH (50 mV/s scan rate) of the 1-butanol-derived GR-MnCo25-Y, Y is (a) 300, (b) 400, and (c) 500. The insets are the reduction potentials of the same CVs.

Figure 11 shows the CVs of the 1-butanol-derived MnCo25-Y (Y = 300, 400, and 500 °C) electrodes. The nearly complete overlap of the 50 consecutive cycles demonstrates their excellent electrochemical stability. The insets emphasize the reduction peaks, whose currents progressively increase with cycling. The redox features assigned to the Mn^3+^/Mn^2+^ (∼ 0.1 V) and Co^3+^/Co^2+^ (∼ 0.4 V) couples indicate the gradual reconstruction of the phosphate framework into mixed (Mn,Co)­(OH)_2_ phases. The comparable magnitudes of the Mn and Co peak currents suggest Co enrichment at the electrode surface resulting from Mn disproportionation and dissolution. In addition, the lower redox currents relative to the corresponding Ni-containing electrodes imply the formation of larger, but more electrochemically active (Mn,Co)­(OH)_2_ particles than the more finely dispersed (Mn,Ni)­(OH)_2_ domains, thus providing similar activity.

The current densities obtained from these electrodes typically reach 100–120 mA cm^–2^ while exhibiting superior electrochemical stability compared with their methanol-derived counterparts. Unlike the ethanol-derived systems, the calcination temperature has only a minor influence on the electrochemical stability of both the methanol- and 1-butanol-derived GR-MnCoX-Y electrodes. The overall cycling behavior and OER performance remain largely unchanged across the investigated temperature range, suggesting that the surface reconstruction processes occurring in alkaline media dominate the electrochemical response. These results further indicate that the formation of *in situ* mixed (Mn,Co)­(OH)_2_/(Mn,Co)­OOH phases govern the catalytic activity, largely diminishing the influence of the initial calcination temperature on long-term electrode stability.

Overall, the methanol-derived electrodes exhibit the highest OER activity, whereas the 1-butanol-derived electrodes provide the best balance between activity and stability. In both solvent systems, increasing the Co content significantly enhances electrode durability, with the MnCo25 composition consistently exhibiting the most stable and active electrochemical performance.

To further evaluate the catalytic efficiency and long-term stability of the electrodes, multichronopotentiometry (m-CP) and chronoamperometry (CA) measurements were performed using the GR-MnMX electrodes. The influence of the secondary transition metal was first investigated using the methanol-derived GR-MnNiX electrodes. Among the compositions examined, the GR-MnNi5 electrode exhibits the lowest Tafel slope, approximately 50 mV dec^–1^, indicating the most favorable OER kinetics. These results demonstrate that even a small amount of Ni incorporation significantly improves the electrocatalytic properties of the LiMnPO_4_ system.^36^ Although the overpotential of the pure GR-LiNiPO_4_ electrode^36^ remains lower than that of the GR-MnNi25 electrode, the introduction of only 5 mol % Ni is sufficient to stabilize the otherwise unstable manganese phosphate electrode while simultaneously promoting faster OER kinetics.

In contrast, the GR-MnNi10 electrode exhibits the highest Tafel slope, approximately 80 mV dec^–1^, indicating slower charge-transfer kinetics during oxygen evolution. Increasing the Ni content to 25 mol % results in a substantial improvement in both stability and catalytic performance. The GR-MnNi25 electrode displays a lower Tafel slope of approximately 64 mV dec^–1^, approaching that of the methanol-derived GR-LiNiPO_4_ electrode.^36^ Furthermore, the overpotentials obtained from the CP measurements are also comparable to those of the pure nickel phosphate electrode.^36^ These observations indicate that Ni incorporation not only suppresses manganese degradation but also enhances long-term OER stability while favorably tuning the reaction kinetics.

The influence of calcination temperature on catalytic activity and durability was further investigated using the graphite-supported 1-butanol-derived GR-MnCo25-Y and ethanol-derived GR-MnNi25-Y electrodes (Figure 12). The CP measurements clearly demonstrate that thermal treatment significantly improves OER performance. For example, the ethanol-derived GR-MnNi25-300 electrode undergoes degradation at current densities above approximately 70 mA cm^–2^. In contrast, the electrode calcined at 500 °C remains stable even at 100 mA cm^–2^ while requiring a lower overpotential to sustain the applied current. These results indicate that the GR-MnNi25–500 electrode is a more efficient and durable OER catalyst than its 300 °C counterpart. A similar trend is observed for the 1-butanol-derived GR-MnCo25-Y electrodes. Among the temperatures investigated, the sample calcined at 500 °C exhibits the most favorable combination of activity and stability. This electrode displays both a relatively low overpotential and a low Tafel slope.

**Figure 12:**
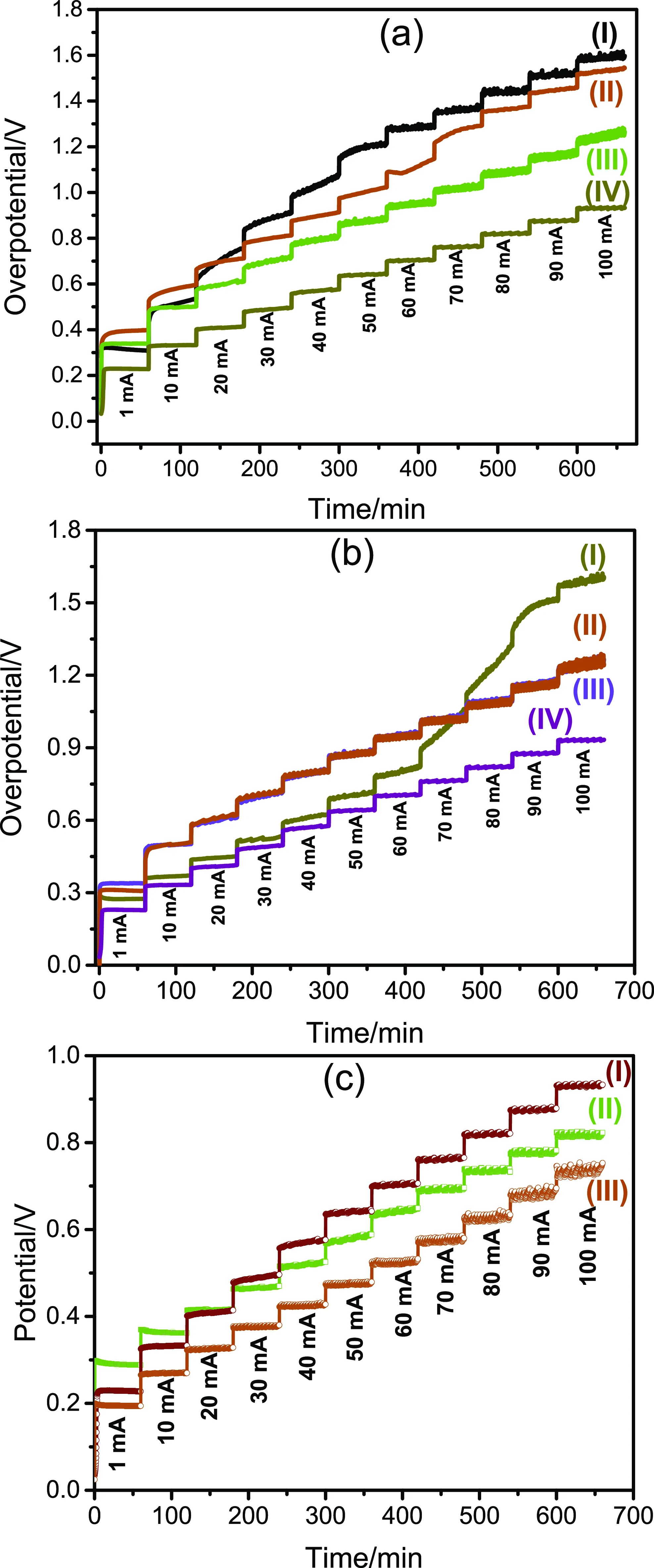
m-CP curves: (a) methanol-derived (I) GR-MnNi5-300, (II) GR-MnNi10-300, (III) GR-MnNi25-300, and (IV) pure Ni-300 electrodes, (b) MnNi25-300 derived from (I) ethanol, (II) 1-butanol, (III) methanol, and (IV) Ni-300 electrode (methanol), and (c) best electrodes (I) GR-MnNi25-300 (methanol), (II) GR-MnCo25-300 (methanol), and (III) GR-MnNi25-500 (ethanol).

The ethanol-derived GR-MnNi25-500 electrode exhibits the best OER performance, requiring overpotentials of only 196 and 819 mV to reach current densities of 1 and 100 mA cm^–2^, respectively. Considering the cell resistance of approximately 4–5 Ω, about 400–500 mV of the overpotential at 100 mA cm^–2^ can be attributed to uncompensated *iR* losses. These findings indicate that increasing the calcination temperature improves the crystallinity of the phosphate framework and, consequently, its resistance to alkaline-induced degradation. As a result, the electrodes calcined at higher temperatures display lower overpotentials, more favorable kinetic parameters, and enhanced long-term stability during OER operation.

Comprehensive multistep chronopotentiometry (m-CP) and chronoamperometry (CA) measurements were performed on the GR-MnM25-Y electrodes (M = Ni or Co), as these compositions exhibited the highest stability among the binary-metal systems. The effects of both the secondary transition metal and the solvent employed during synthesis on the OER activity and durability were systematically investigated, and the results are summarized in Table 2. Among the examined electrodes, the ethanol-derived GR-MnNi25-300 electrode displays the lowest Tafel slope, indicating the most favorable OER kinetics. Despite its high intrinsic activity, this electrode exhibits limited stability at elevated current densities and undergoes degradation during prolonged operation, as evidenced by the m-CP measurements. In contrast, annealing the same composition at 500 °C significantly enhances its structural stability while maintaining high catalytic activity, resulting in the best overall OER performance among the investigated electrodes.

**Table 2:** Tafel Slopes of GR-MnM25-300 (M = Ni and Co) Electrodes

GR-Electrodes	Solvent	Tafel Slopes (mV/dec)	GR-Electrodes	Solvent	Tafel Slope (mV/dec)
GR-Ni-300	Methanol	69	GR-Co-300	Methanol	47
GR-MnNi25-300	Methanol	64	GR-MnCo25-300	Methanol	75
GR-MnNi25-300	Ethanol	54	GR-MnCo25-300	Ethanol	59
GR-MnNi25-300	1-Butanol	61	GR-MnCo25-300	1-Butanol	52

## Conclusion

The effects of solvent (methanol, ethanol, and 1-butanol) on the self-assembly of lithium nitrate–manganese nitrate tetrahydrate–metal nitrate hexahydrate (M = Ni, Co)–phosphoric acid–Pluronic P123 systems were systematically investigated in their solution, gel, and mesostructured semisolid phases for the fabrication of mesoporous LiMn_1–x_M_x_PO_4_ electrodes. In ethanol and 1-butanol media, a fraction of Mn­(II) undergoes oxidation and precipitates as crystalline MnPO_4_·H_2_O, whereas this process is considerably suppressed in methanol for both Ni- and Co-containing systems. The extent of precipitation is more pronounced in the Co-containing compositions than in the corresponding Ni-containing systems. Upon calcination, the precipitated MnPO_4_·H_2_O undergoes thermal reduction and condensation reactions to form Mn_2_P_2_O_7_.

The solution phases predominantly yield olivine-type LiMn_1–x_M_x_PO_4_, provided that MnPO_4_·H_2_O precipitation remains limited. More extensive precipitation alters the solution stoichiometry and results in mixed-phase products. In contrast, both the methanol-derived solutions and their supernatant fractions consistently produce phase-pure olivine LiMn_1–x_M_x_PO_4_. In all cases, Ni­(II) and Co­(II) are homogeneously incorporated into the olivine lattice, indicating true solid-solution behavior without phase segregation.

Both solution and supernatant fractions can be readily processed into electrodes by spin coating on FTO substrates or dip coating on graphite rods. Although FTO-supported electrodes exhibit poor long-term stability under alkaline OER conditions, graphite-supported electrodes display excellent mechanical and electrochemical robustness. Irrespective of their initial composition, all LiMn_1–x_M_x_PO_4_ electrodes undergo complete in situ reconstruction to mixed-metal hydroxides in alkaline media, demonstrating that the reconstructed hydroxide phases are the true catalytically active species during OER.

The incorporation of either Ni or Co significantly improves the stability of manganese-based electrodes, even at a substitution level of only 5 mol %, while electrodes containing 25 mol % secondary metal exhibit outstanding robustness and enhanced OER performance. The synthesis solvent also strongly influences the electrochemical behavior of the reconstructed electrodes. In general, methanol- and 1-butanol-derived electrodes display superior operational stability, and increasing the secondary-metal content simultaneously enhances both electrode durability and OER activity.

For the Ni-containing systems, the ethanol-derived GR-MnNi25 electrode exhibits the lowest Tafel slope and the fastest OER kinetics. However, the methanol- and 1-butanol-derived GR-MnNi25 electrodes show markedly improved stability over a broad current-density range. Although their catalytic activities are slightly lower than those of the ethanol-derived electrode, their substantially enhanced durability renders them more attractive for long-term operation. The improved stability is likely associated with solvent-induced differences in morphology, crystallinity, surface nickel distribution, and the reconstruction pathways of the mixed-hydroxide phases.

These findings highlight the inherent trade-off between catalytic activity and durability in the Ni-containing systems. While ethanol-derived electrodes provide the highest initial OER activity, methanol- and 1-butanol-derived electrodes offer a more favorable balance between activity and long-term stability. Therefore, solvent selection plays a critical role in determining not only the initial electrocatalytic performance but also the operational lifetime of the reconstructed mixed-metal hydroxide catalysts under alkaline OER conditions.

For the Co-containing systems, Co incorporation likewise substantially enhances the stability of LiMnPO_4_-derived electrodes. The methanol-derived GR-MnCo25-300 electrode exhibits the highest Tafel slope and comparatively slower OER kinetics, whereas the 1-butanol-derived GR-MnCo25 electrode displays lower overpotentials and superior catalytic activity. Overall, the incorporation of either Ni­(II) or Co­(II) into LiMnPO_4_ provides a viable strategy for stabilizing manganese-based electrodes and improving their OER performance through the formation of robust, catalytically active mixed-metal hydroxide phases generated *in situ* under alkaline conditions.

## Supplementary Material



## Notes

Tables for compositions and XRD analysis, schematic representations for electrode preparation, XRD patterns and ATR-FTIR spectra of the gel-phase and calcined samples, XPS spectra and SEM images, photograph of the electrochemical set up, and CVs for FTO and GR electrodes (PDF)

## Abbreviations

OER: oxygen evolution reaction
CP: chronopotentiometry
CA: chronoamperometry
BET: Brunauer-Emmett-Teller
BJH: Barrett–Joyner–Halenda.
