# Supplementary material for: Mesoporous LiMn1–xMxPO4 (M = Ni, Co) Precatalysts by Alcohol-Mediated Self-Assembly: Hydroxide Reconstruction and Suppression of Manganese Degradation for Stable Alkaline Oxygen Evolution
Source: Inorg Chem. 2026 Aug 18;65(34):20104–21. doi: 10.1021/acs.inorgchem.6c03469 (PMC13536938; doi:10.1021/acs.inorgchem.6c03469)
Supplement: Supplementary file 1 [file ic6c03469_si_001.pdf]

Supporting Information

Mesoporous $LiMn_{1-x}M_xPO_4$ (M = Ni, Co) Pre-catalysts by Alcohol-Mediated Self-Assembly: Hydroxide Reconstruction and Suppression of Manganese Degradation for Stable Alkaline Oxygen Evolution

Arda Altan[a], Irmak Karakaya Durukan[a], and Ömer Dag[a,b]*

[a]Department of Chemistry, Bilkent University, 06800, Ankara, Turkey
[b]UNAM — National Nanotechnology Research Center and Institute of Materials Science and Nanotechnology, Bilkent University, 06800, Ankara, Turkey.
Email: dag@fen.bilkent.edu.tr

Table S1. Amounts of chemicals in gram, used for the solutions with mole ratio of $1P123{:}30LiNO_3{:}30M(NO_3)_2{\cdot}nH_2O$ (M is mixture Mn(II) and secondary metal (Ni or Co):$30H_3PO_4$ in all solvents (ethanol, methanol, and 1-butanol).

**Solutions**	**P123**	**$LiNO_3$**	**$M(NO_3)_2{\cdot}6H_2O$**	**$Mn(NO_3)_2{\cdot}4H_2O$**	**H_3PO_4**	**Solvent**
LMnNi5P	1	0.357	0.076	1.234	0.596	10
LMnNi10P	1	0.357	0.151	1.169	0.596	10
LMnNi25P	1	0.357	0.376	0.974	0.596	10
LMnCo5P	1	0.357	0.076	1.234	0.596	10
LMnCo10P	1	0.357	0.151	1.169	0.596	10
LMnCo25P	1	0.357	0.376	0.974	0.596	10

Table S2. Lattice parameters a, b, c, and volume of the Pmnb orthorhombic olivine-phase of ethanol-driven LMnP, LMnCo25P, and LCoP calcined at 700°C.

Samples	a (Å)	b (Å)	c (Å)	Volume ($Å^3$)
LMnP-E-700	6.092	10.377	4.724	298.6
LMnCo25P-E-700	6.032	10.310	4.703	292.5
LCoP-E-700	5.908	10.125	4.675	279.6

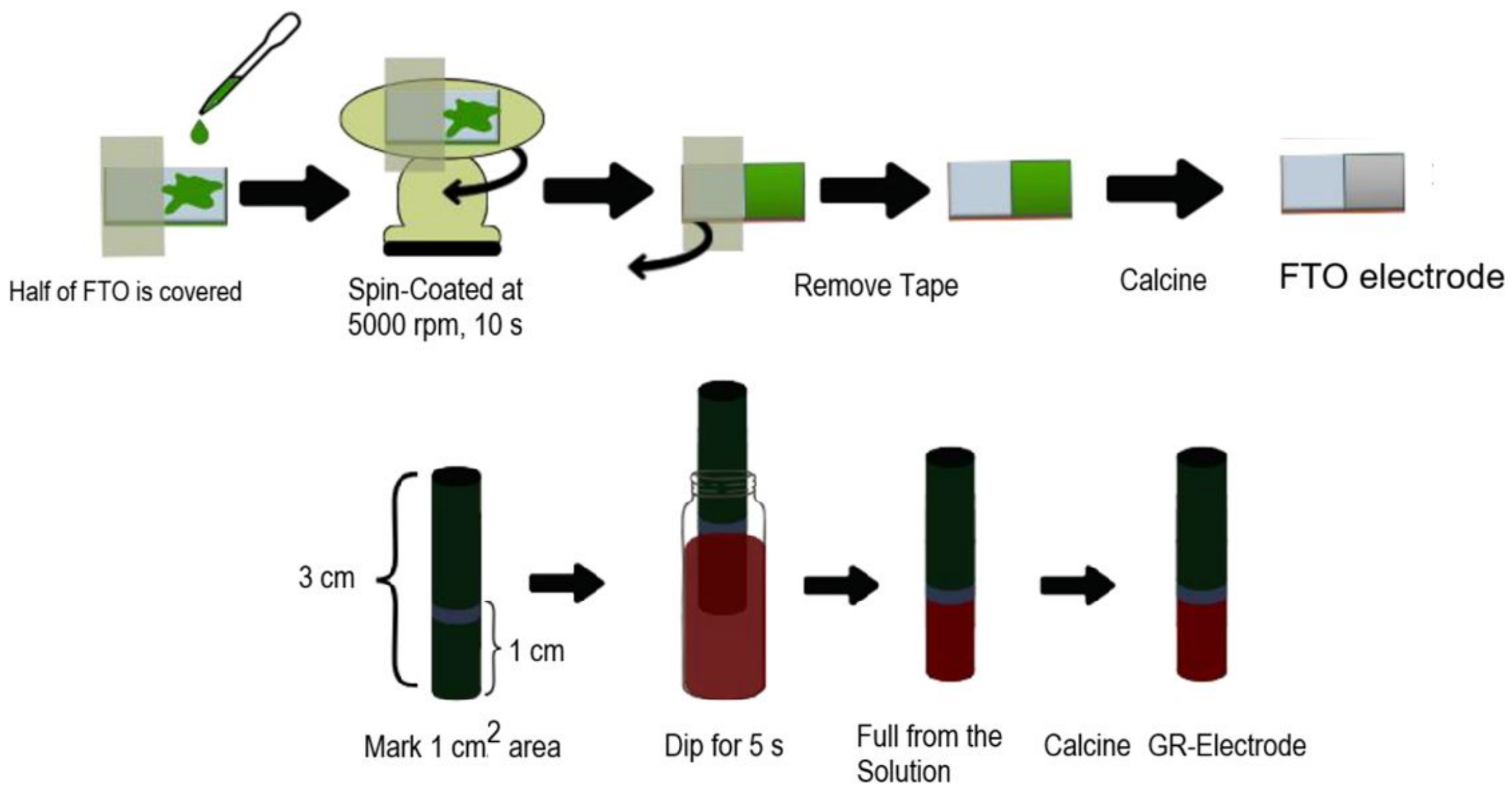

Figure S1. Schematic representation of the fabrication process of the electrodes.

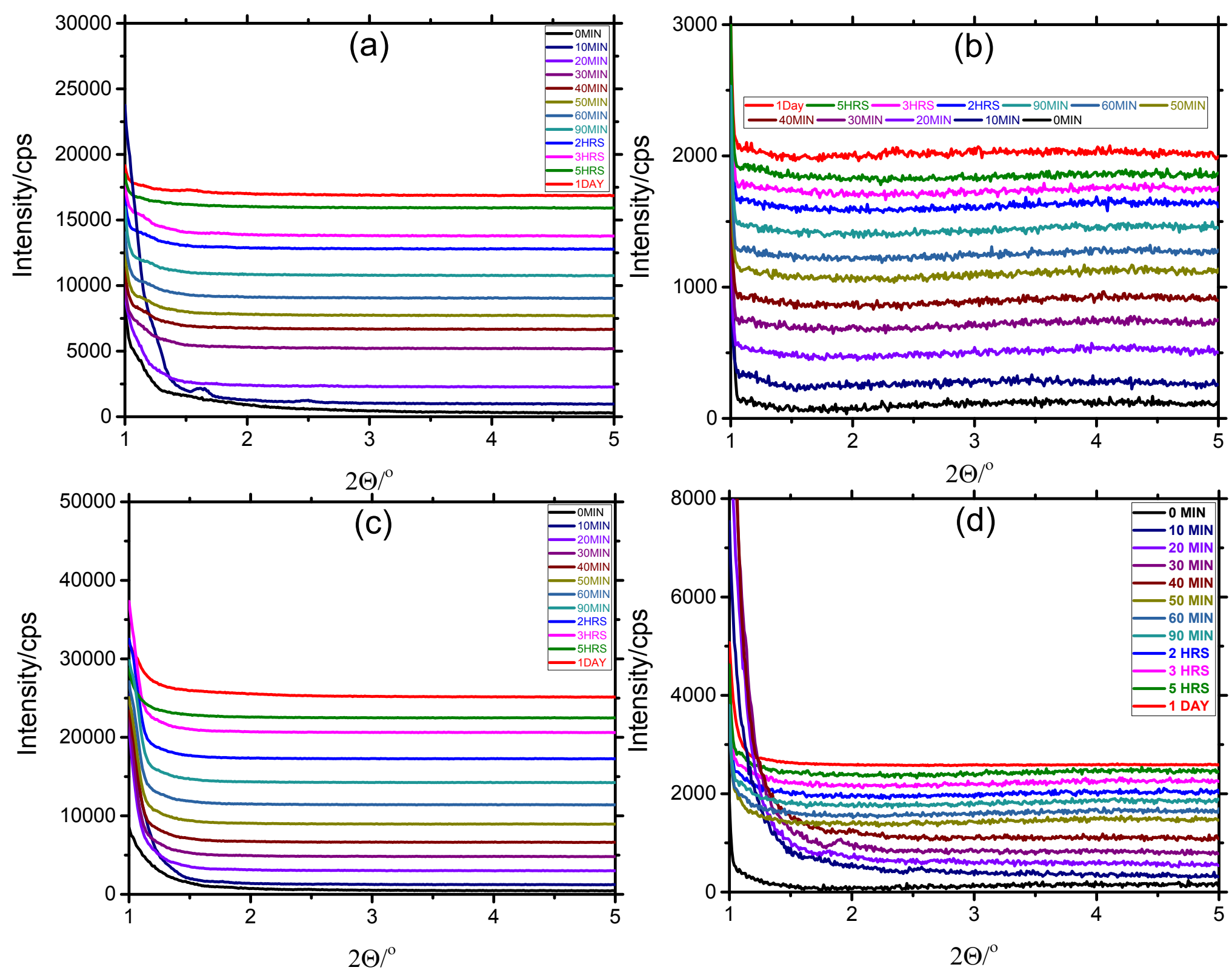

Figure S2. Small-angle XRD patterns: the LMnNi10P and LMnNi25P from supernatants of ethanol (a, c) and methanol (b, d), during gelation.

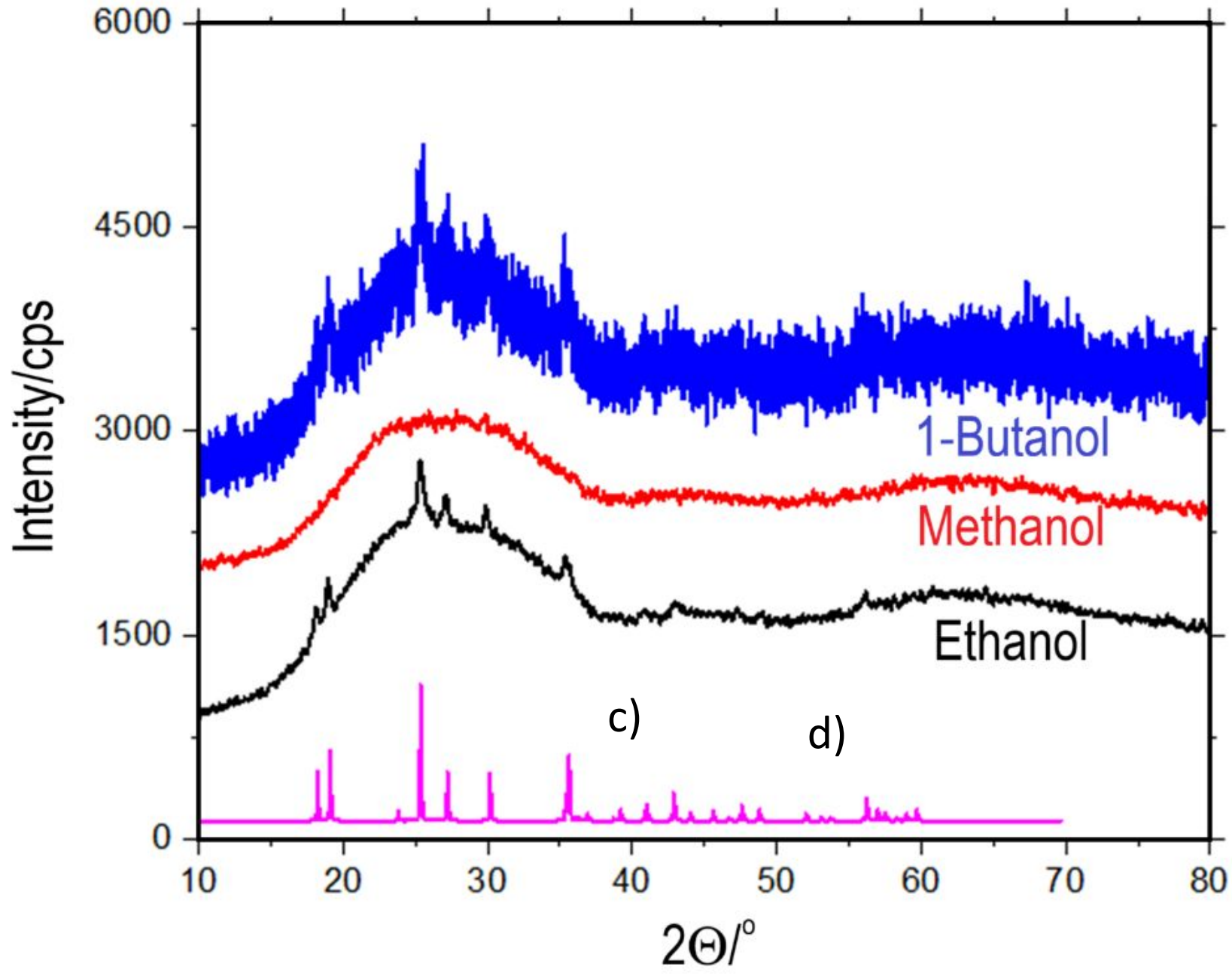

Figure S3. Small-angle XRD patterns of the LMnNi25P solutions of ethanol, methanol, and 1-butanol with the reference ICDD PDF-Card of $MnPO_4 \cdot H_2O$ (00-044-0071).

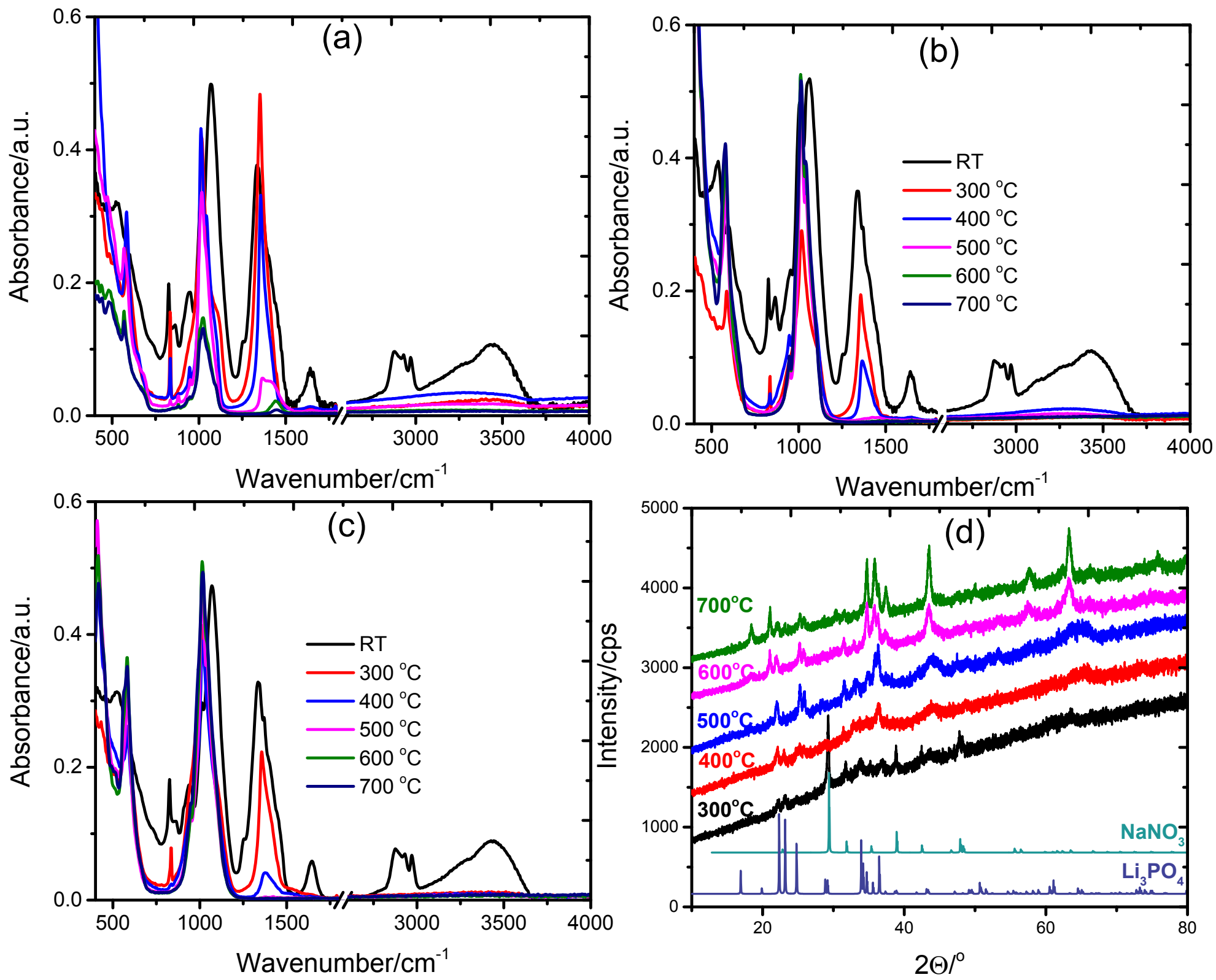

Figure S4. Temperature dependent ATR-FTIR spectra of ethanol derived (a) MnNi5-Y, (b) MnNi10-Y, c) MnNi25-Y samples and d) the XRD patterns of MnNi25-Y with ICDD PDF cards of $NaNO_3$ (00-036-1474) and Li_3PO_4 (00-015-0760).

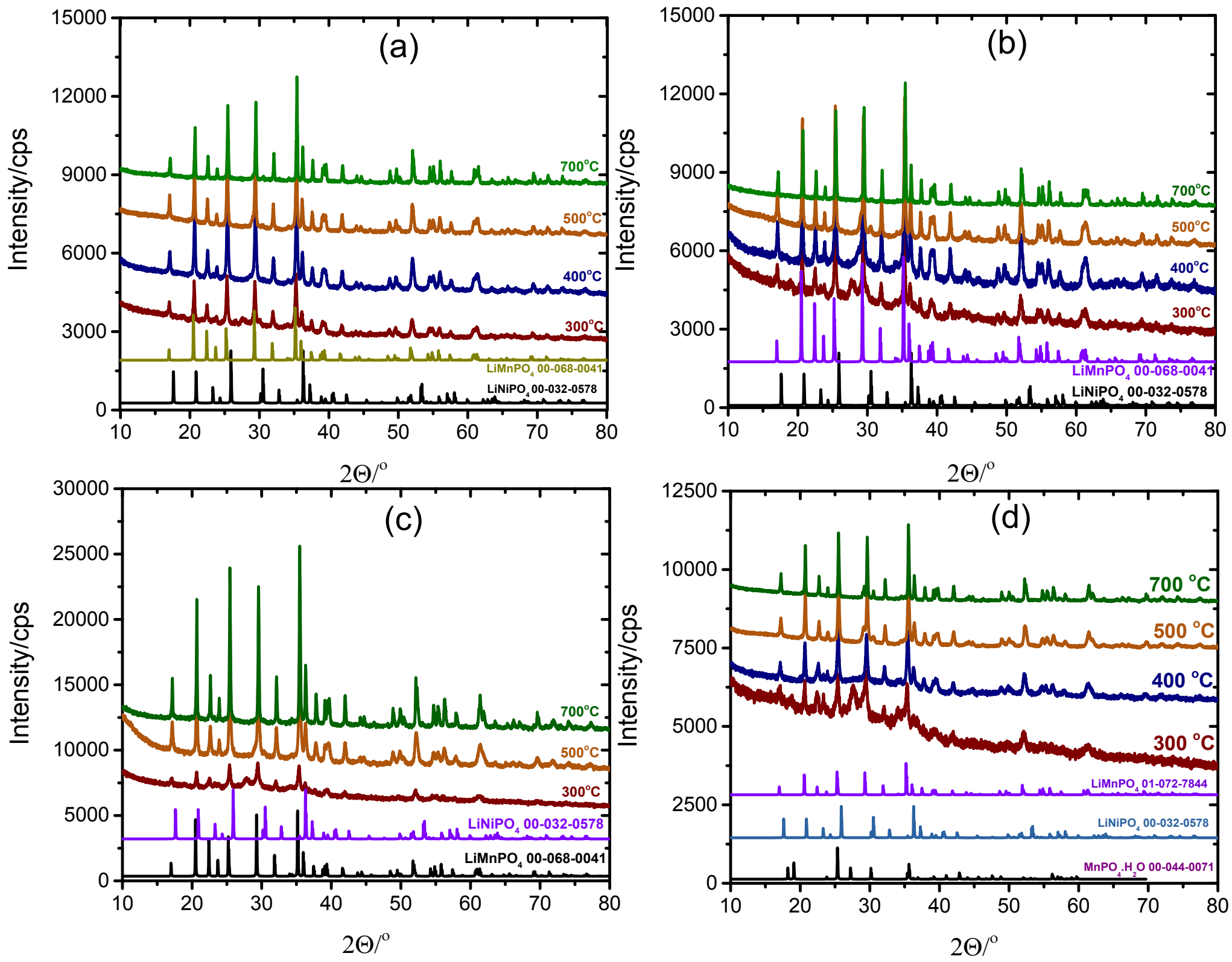

Figure S5. XRD patterns of the calcined $LiMn_{1-x}Ni_xPO_4$ methanol solutions with x of (a) 0.05, (b) 0.10, and (c) 0.25, and (d) $LiMn_{0.75}Ni_{0.25}PO_4$ ethanol solution with ICDD PDF cards of $LiMnPO_4$ (00-068-0041), $LiNiPO_4$ (00-032-0578), and $MnPO_4 \cdot H_2O$ (00-044-0071).

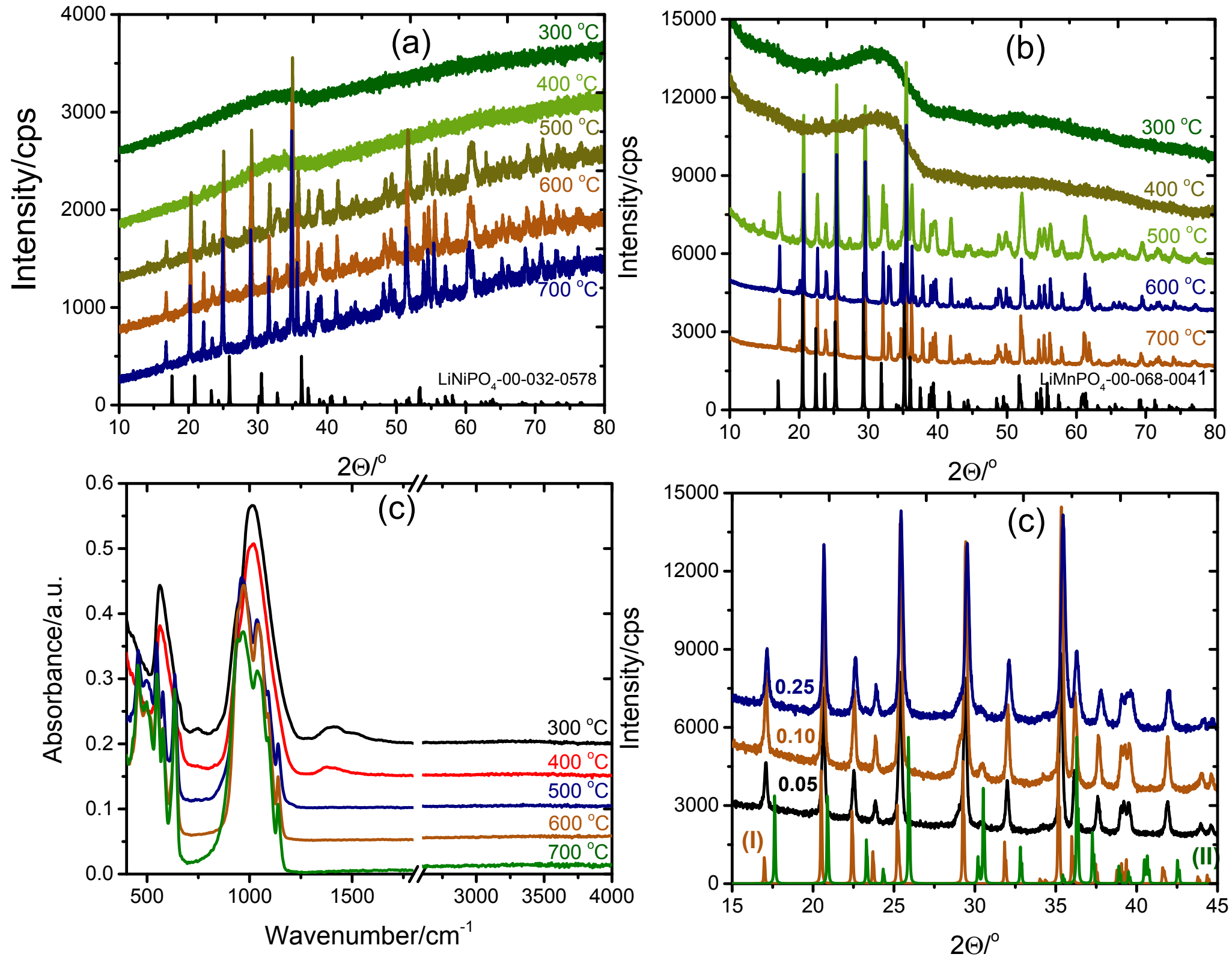

Figure S6. Methanol supernatant: XRD patterns of $LiMn_{1-x}Ni_xPO_4$, where x is (a) 0.05 and (b) 0.25 and (c) ATR-FTIR spectra of $LiMn_{0.75}Ni_{0.25}PO_4$. (d) Comparison of the XRD patterns of $LiMn_{1-x}Ni_xPO_4$ (x= 0.05, 0.10, and 0.25) samples from methanol solutions, calcined at 500°C with ICDD PDF cards of $LiNiPO_4$ (00-032-0578) and $LiMnPO_4$ (00-068-0041).

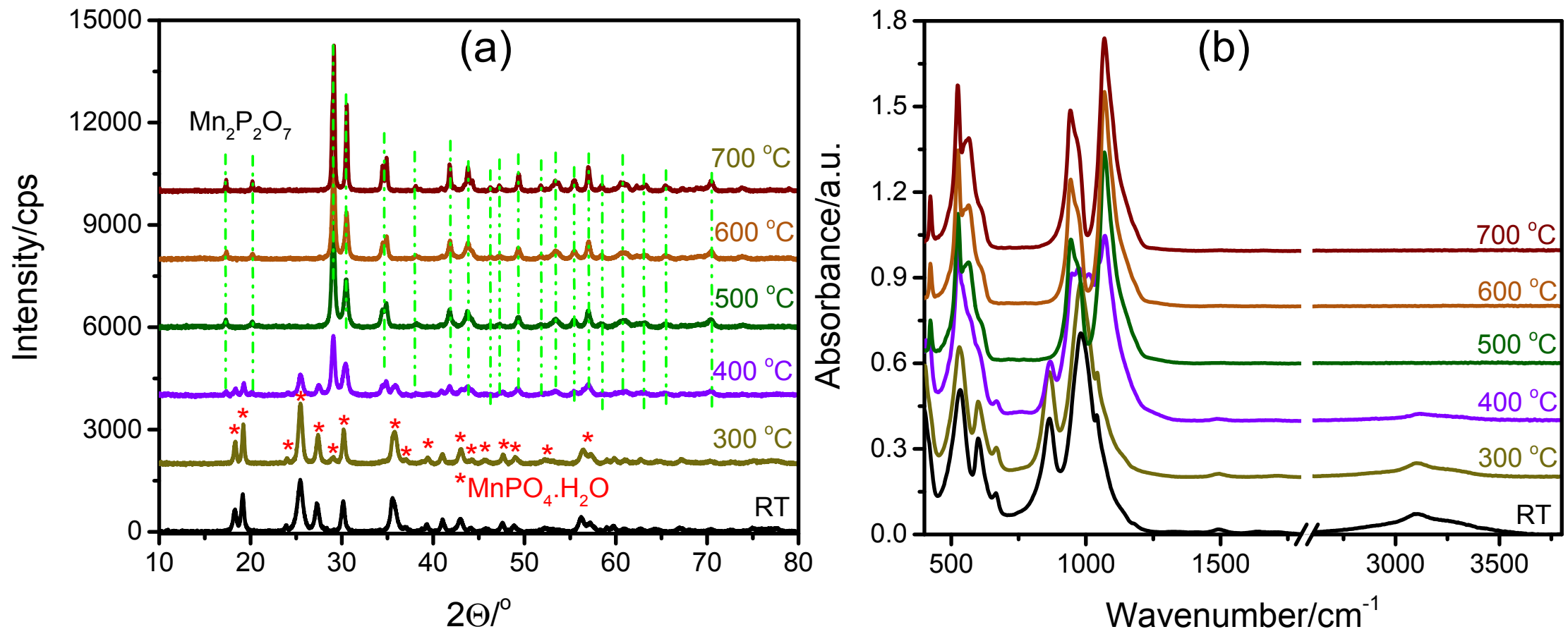

Figure S7. Temperature dependent (a) wide-angle XRD patterns and (b) ATR-FTIR spectra of the MnCo5 precipitates from ethanol solutions.

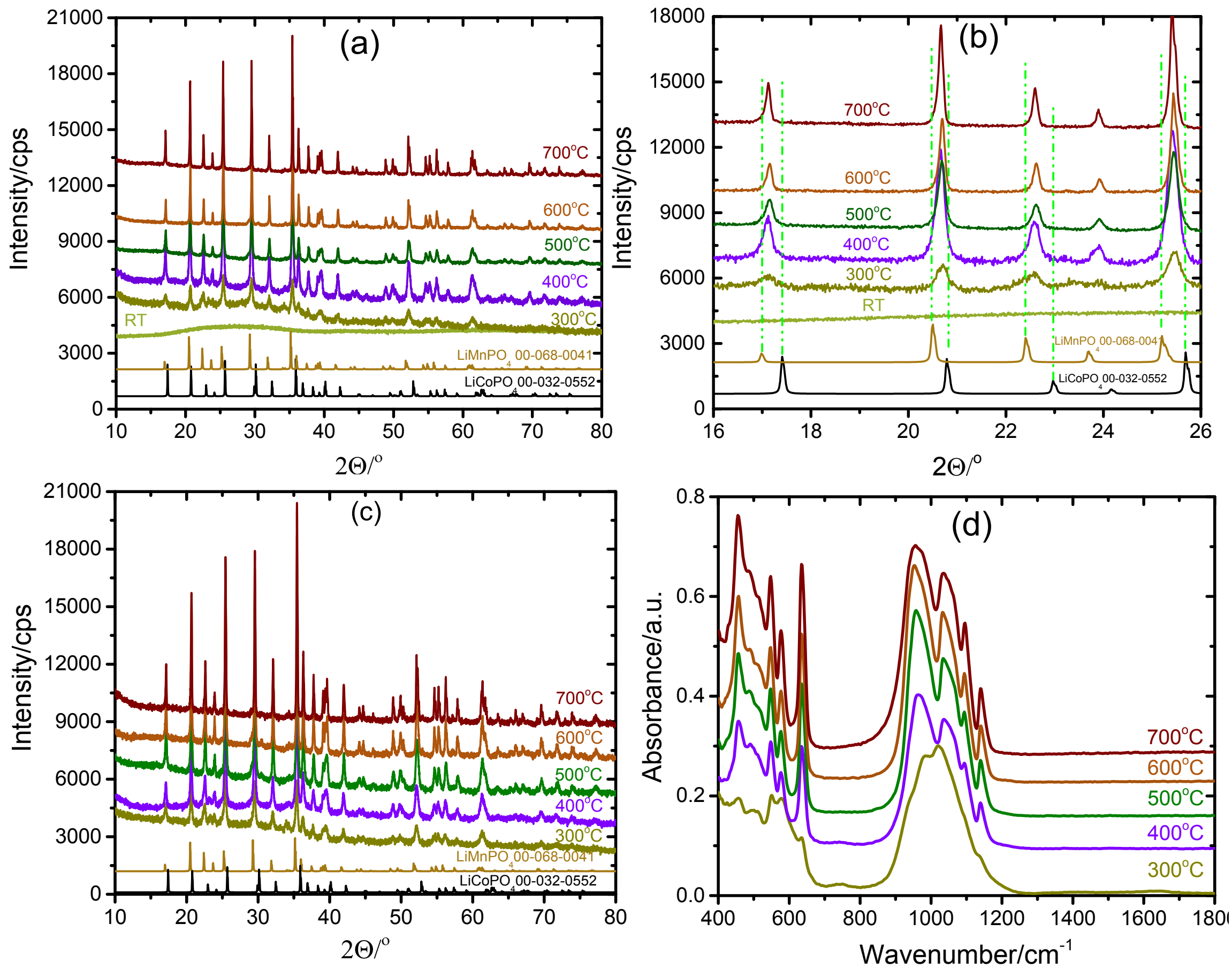

Figure S8. XRD patterns of the calcined (a) and (b) solution, (c) supernatant, and (d) ATR-FTIR spectra of the MnCo25-Y from methanol solution.

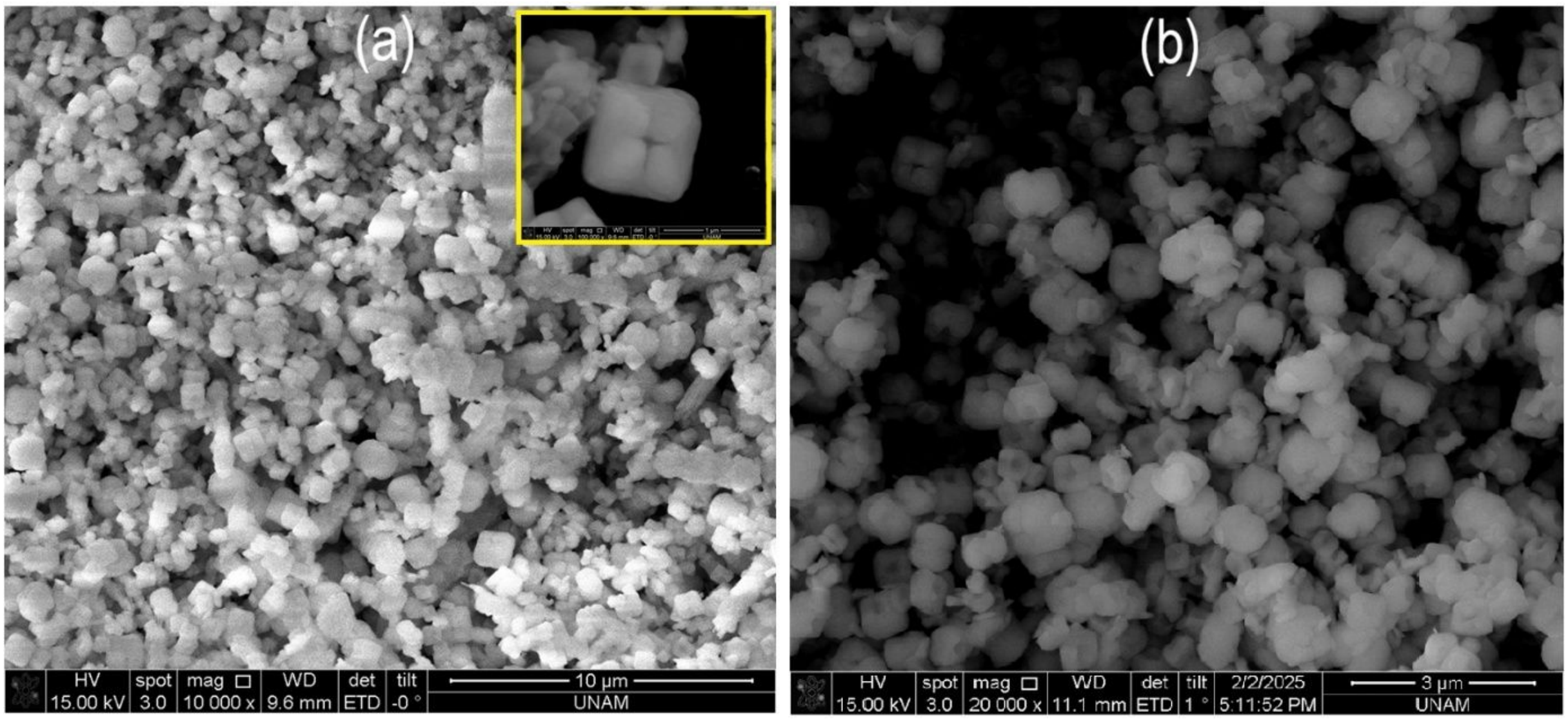

Figure S9. SEM image of the precipitate from (a) ethanol (inset magnified image, scale bar 1 μm) and (b) 1-butanol solutions.

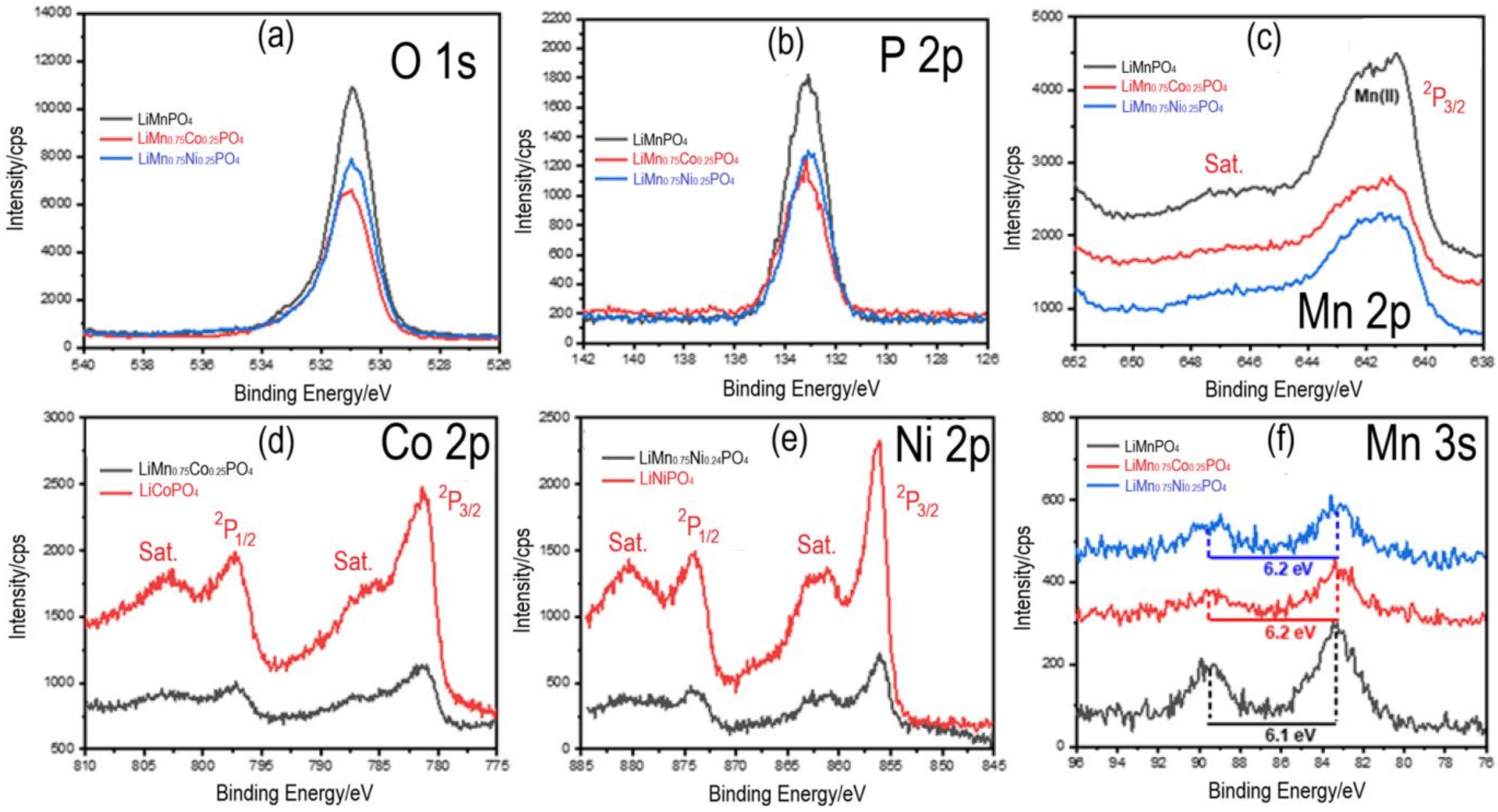

Figure S10. High resolution XPS spectra of the MnNi25-300 and MnCo25-300 samples (derived from methanol) in the O 1s, P 2p, Mn 2p, Co 2p, Ni 2p, and Mn 3s regions.

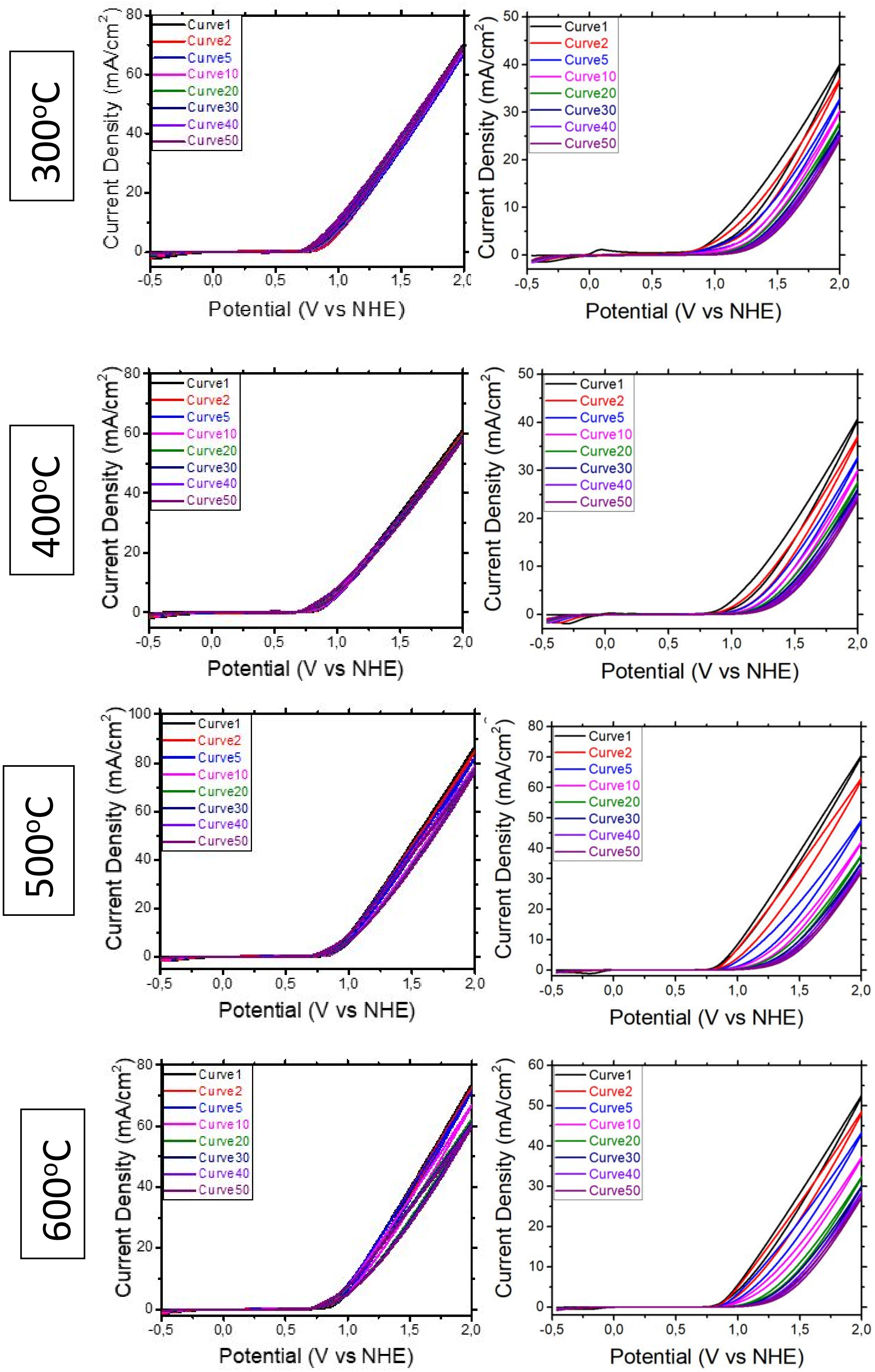

Figure S11. Selected CVs (1st, 2nd, 5th, 10th, 20th, 30th, 40th, and 50th) of 50 CV cycles at a 50mV/s scan rate of the FTO-MnNi5-Y and FTO-MnCo5-Y electrodes, derived from ethanol solution.

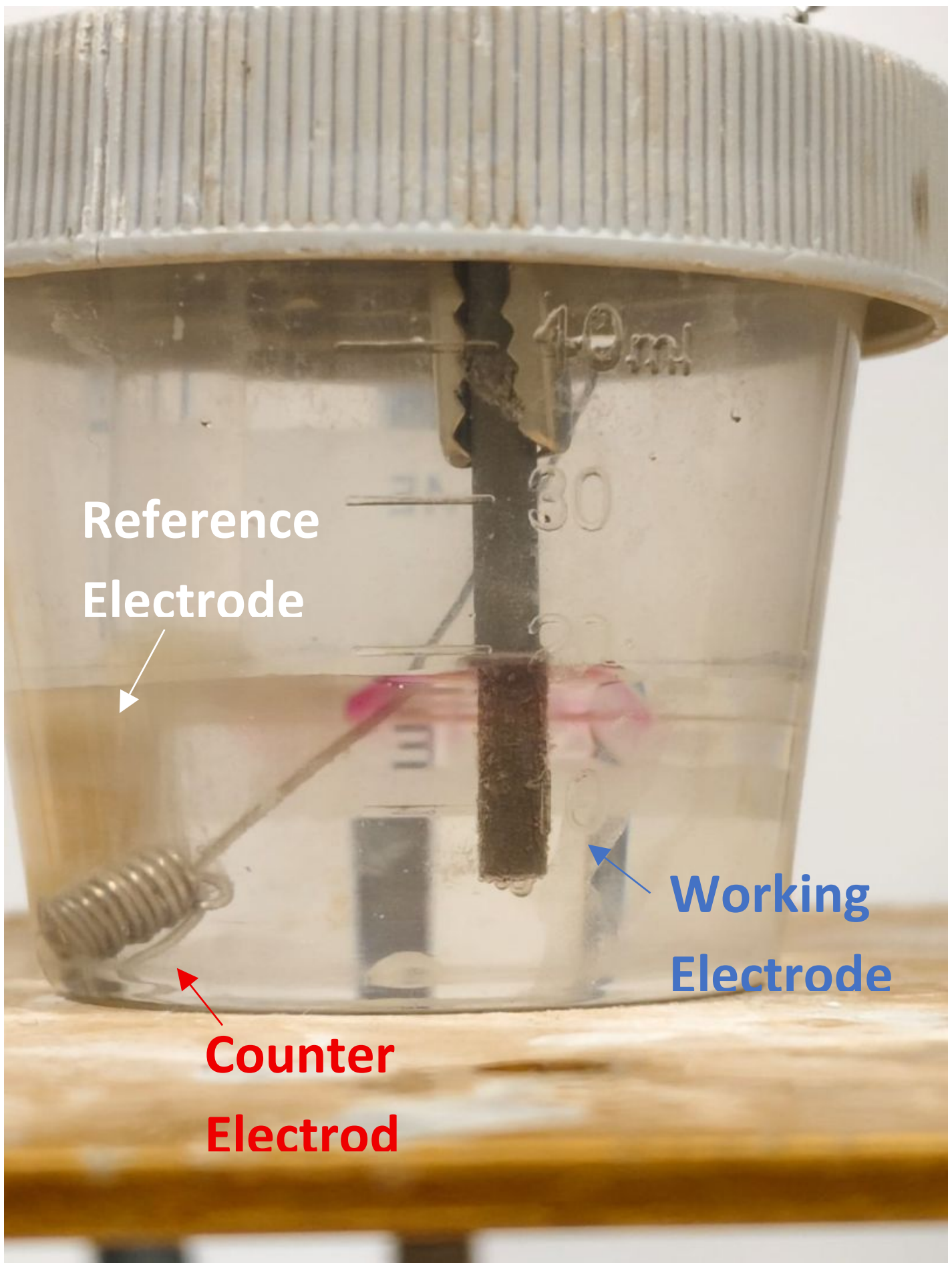

Figure S12. Photograph of electrolyte solution during OER in 1 M KOH solution.

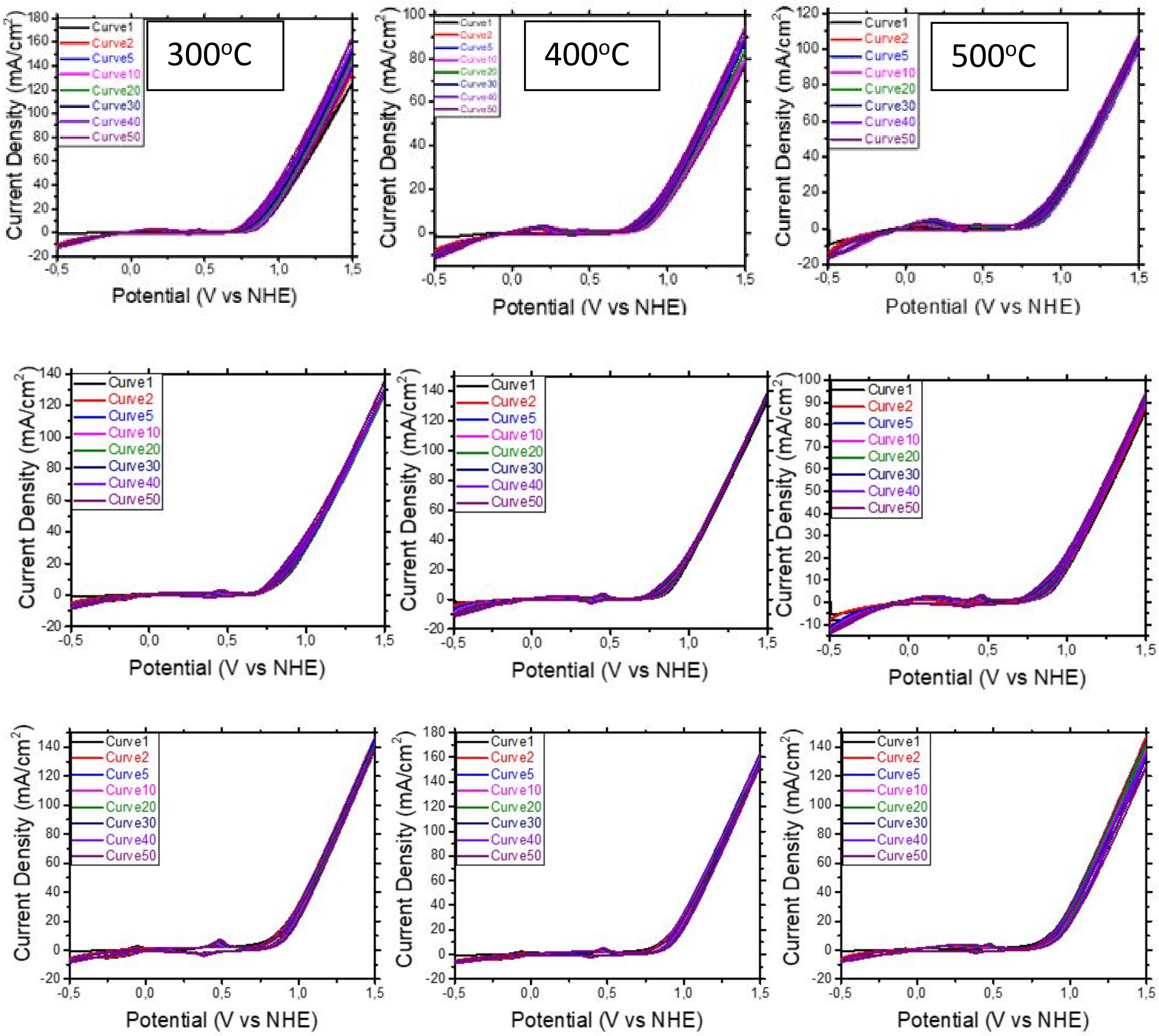

Figure S13. Selected CVs (1st, 2nd, 5th, 10th, 20th, 30th, 40th, and 50th) of 50 CV cycles at scan rate of 50mV/s of the ethanol-derived GR-MnNi5-Y (1st row), GR-MnNi10-Y (2nd row), and GR-MnNi25-Y (3rd row) electrodes, calcined at 300 (1st column), 400 (2nd clumn), and 500 oC(3rd column).

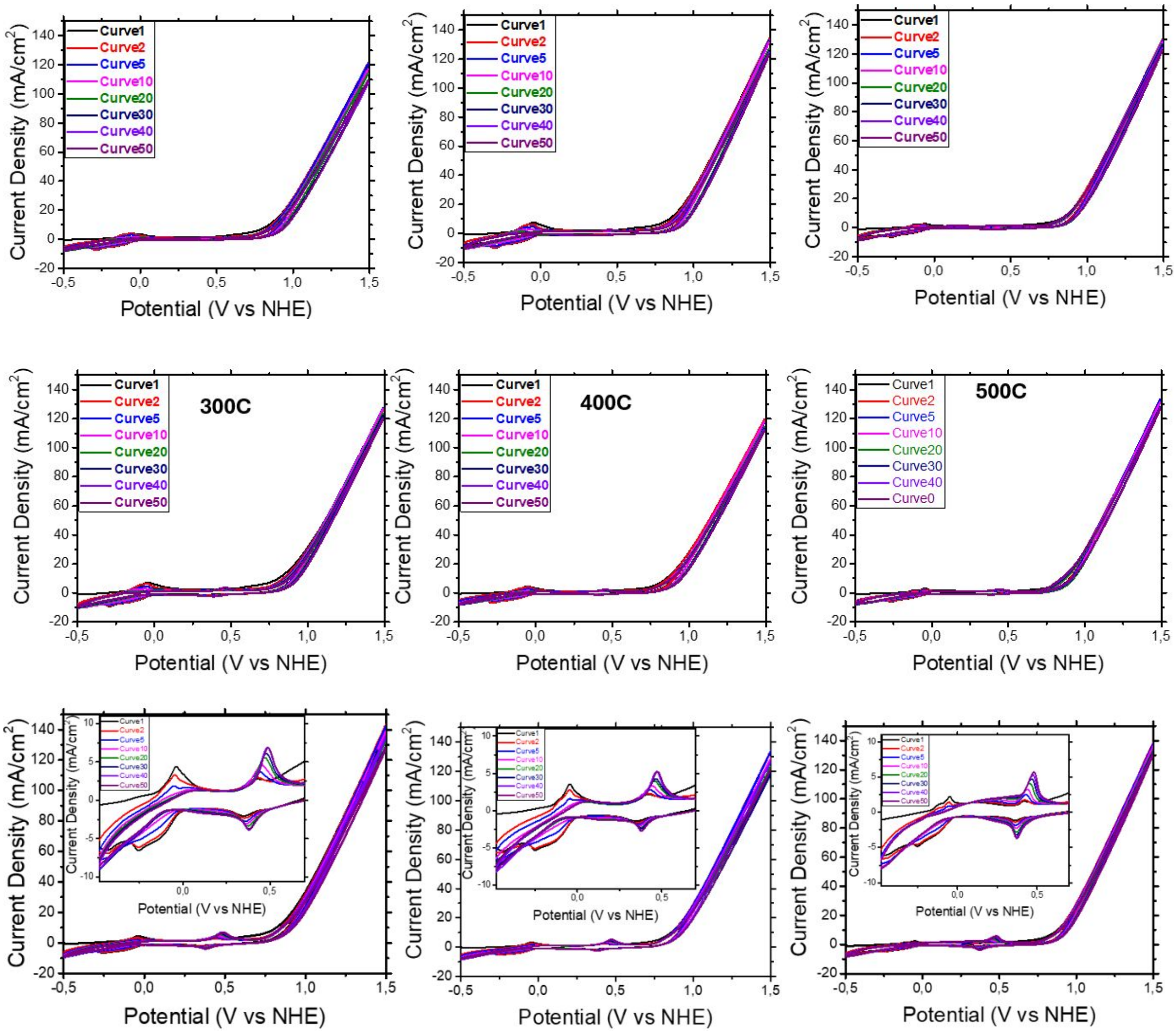

Figure S14. Selected CVs (1st, 2nd, 5th, 10th, 20th, 30th, 40th, and 50th) of 50 CV cycles with a scan rate of 50 mV/s of the methanol-derived GR-MnNi5-Y (1st row), GR-MnNi10-Y (2nd row), and GR-MnNi25-Y (3rd row) electrodes, calcined at 300 (1st column), 400 (2nd column), and 500 oC (3rd column). Insets are the CVs in to redox region.

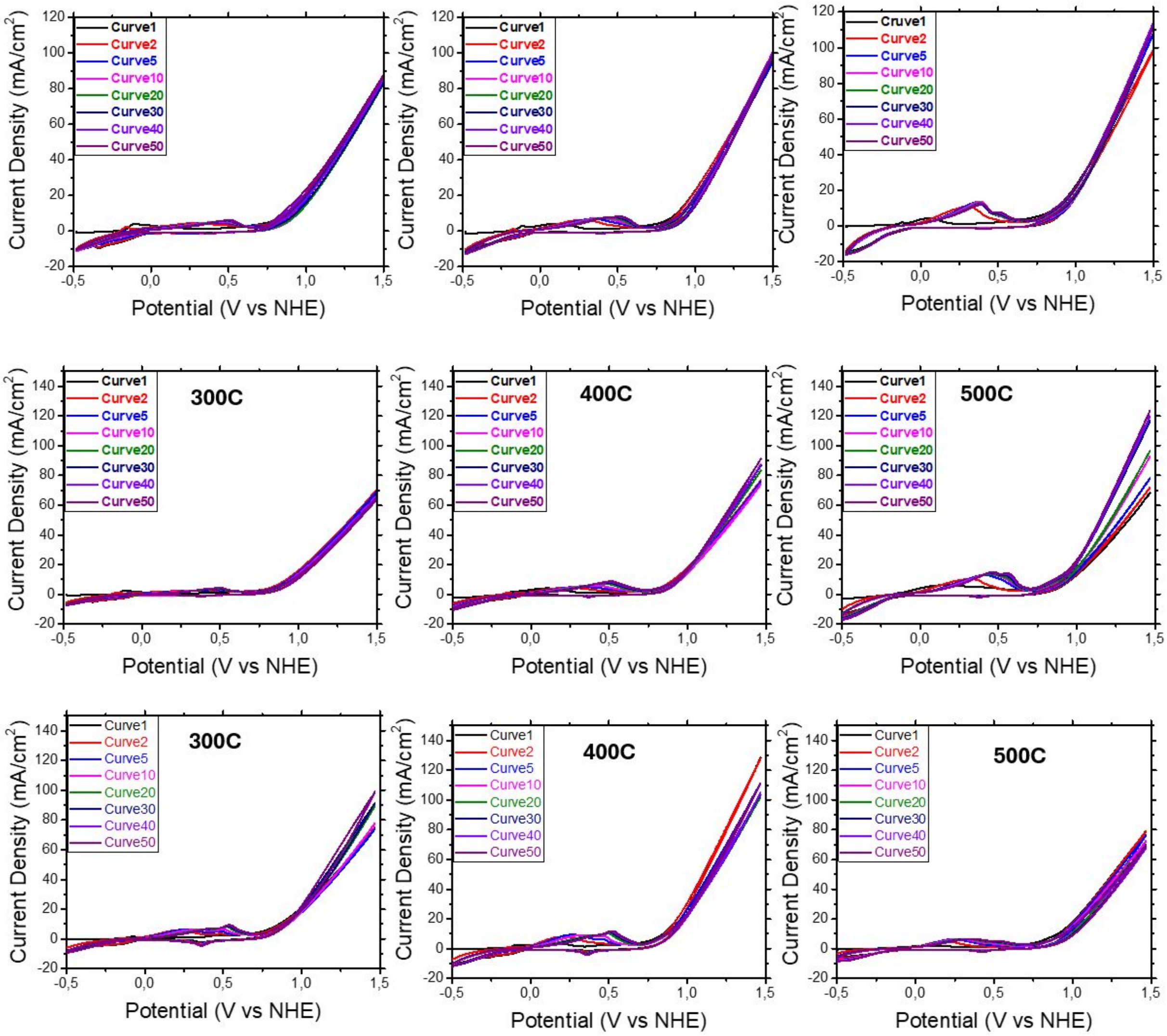

Figure S15. Selected CVs (1st, 2nd, 5th, 10th, 20th, 30th, 40th, and 50th) of 50 CV cycles with a scan rate of 50 mV/s of 1-butanol-derived GR-MnNi5-Y (1st row), GR-MnNi10-Y (2nd row), and GR-MnNi25-Y (3rd row) electrodes at 300 (1st column), 400 (2nd column), and 500 °C (3rd column).

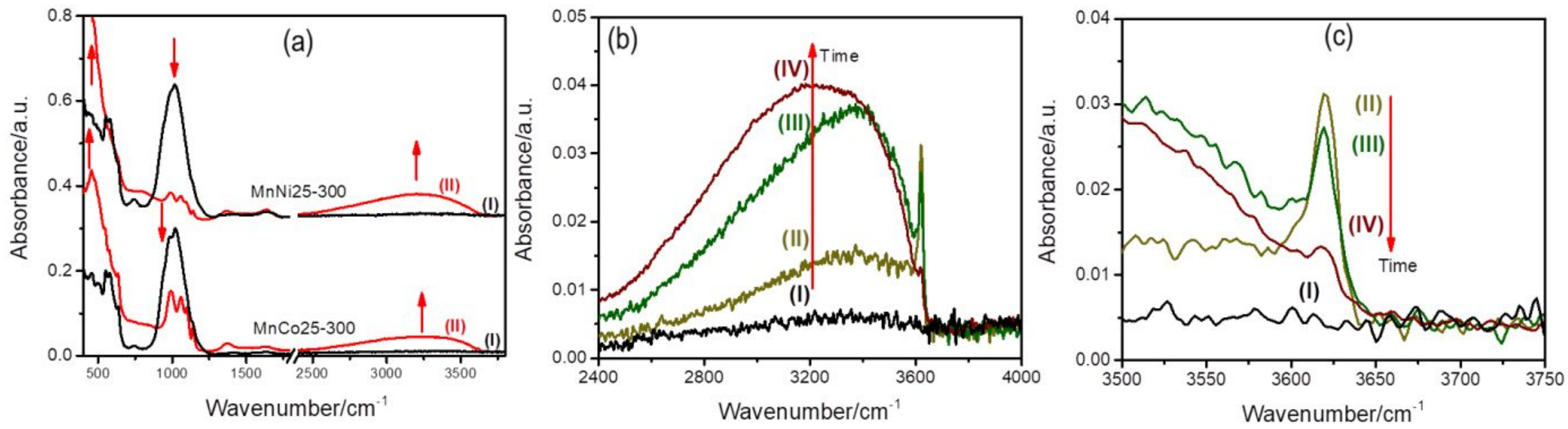

Figure S16. ATR-FTIR spectra of (a) MnNi25-300 (I) before and (II) after keeping 3 h in 1 M KOH solution and MnCo25-300 (I) before and (II) after keeping 1 h in 1 M KOH solution, (b and c) MnCo25-300 (I) before and after keeping (II) 15 min, (III) 30 min, and (IV) 1 h in 1 M KOH solution.

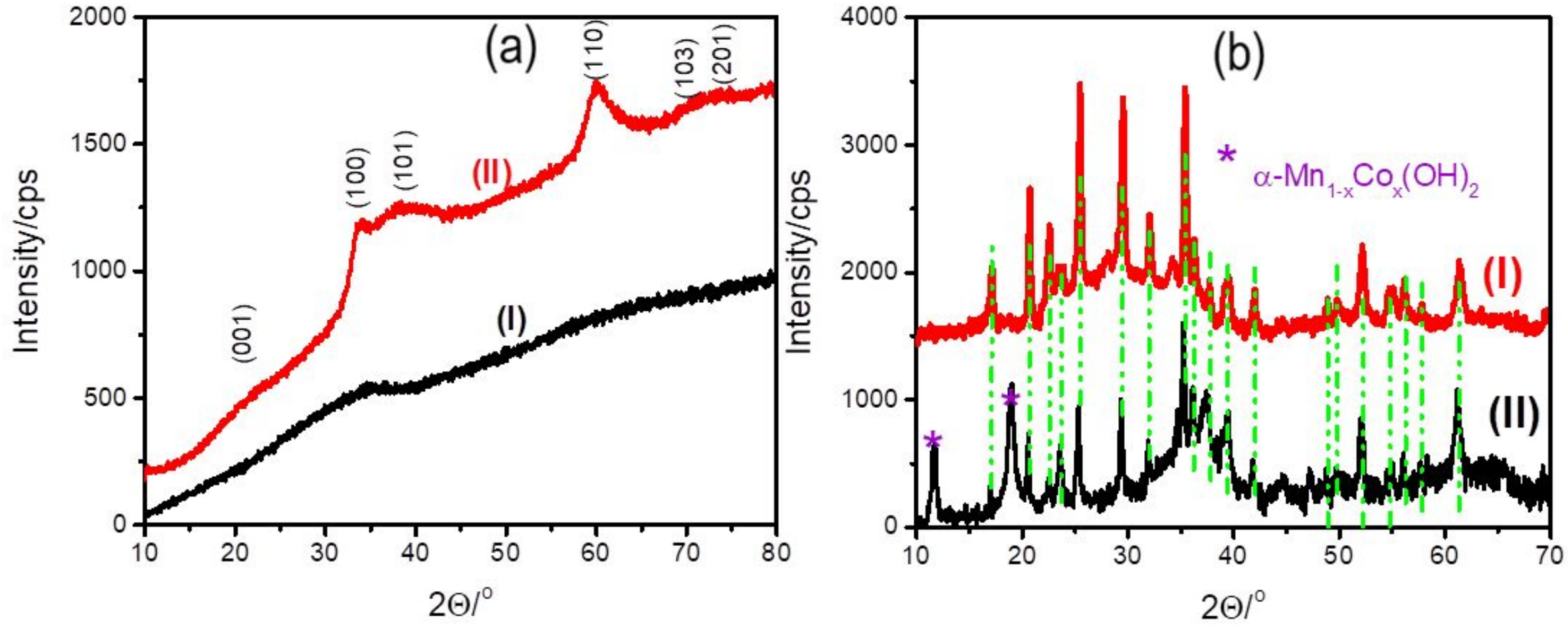

Figure S17. XRD patterns of (a) MnNi25-300 (I) before and (II) after keeping 3 h in 1 M KOH solution and (b) MnCo25-300 (I) before and (II) after keeping 1 h in 1 M KOH solution.

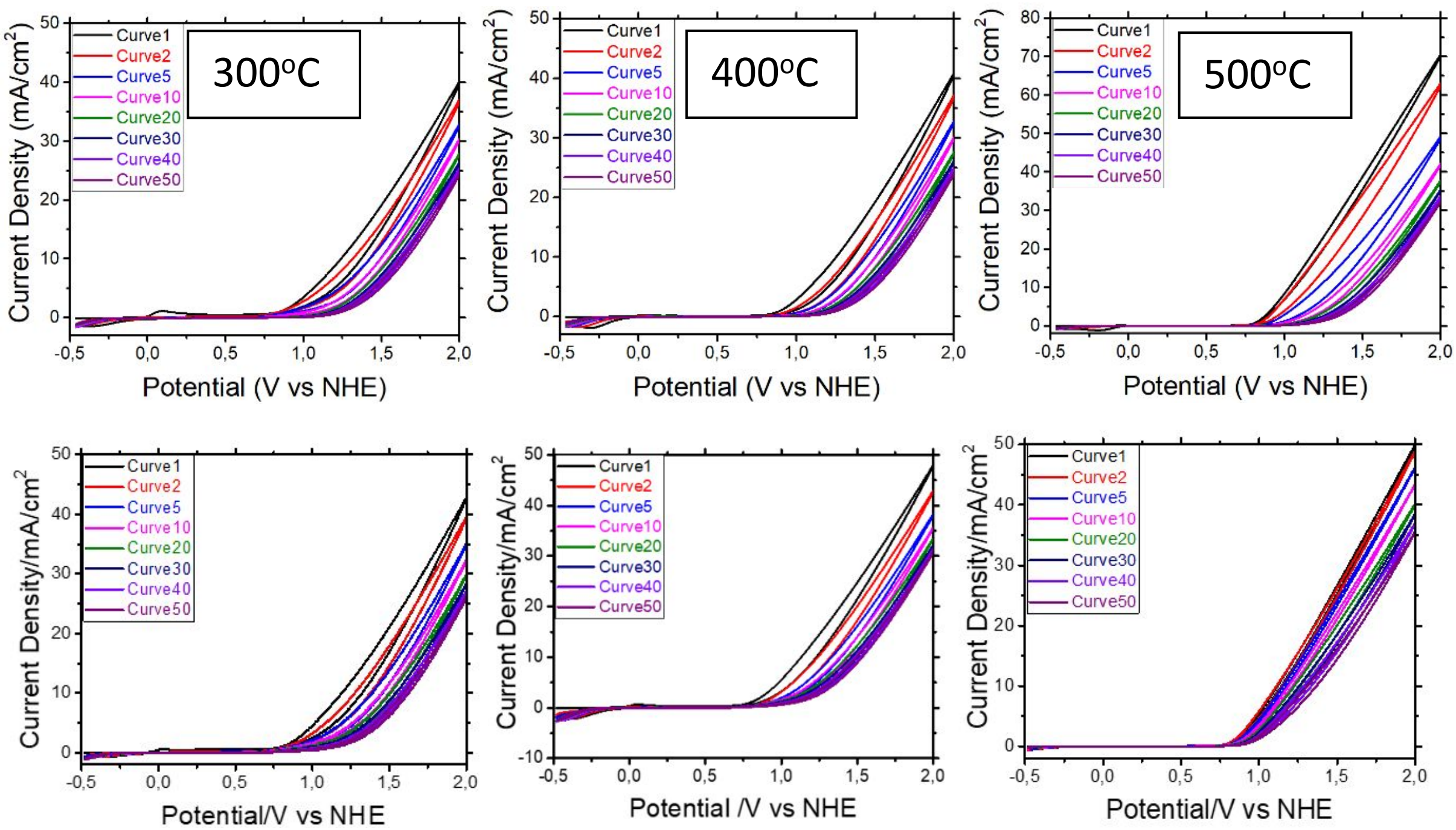

Figure S18. Selected CVs (1st, 2nd, 5th, 10th, 20th, 30th, 40th, and 50th) of 50 CV cycles in 1 M KOH of the ethanol-derived FTO-MnCo5-Y(1st row) and FTO-MnCo10-Y(2nd row), calcined at (left to right) 300 to 500 ^{o}C.

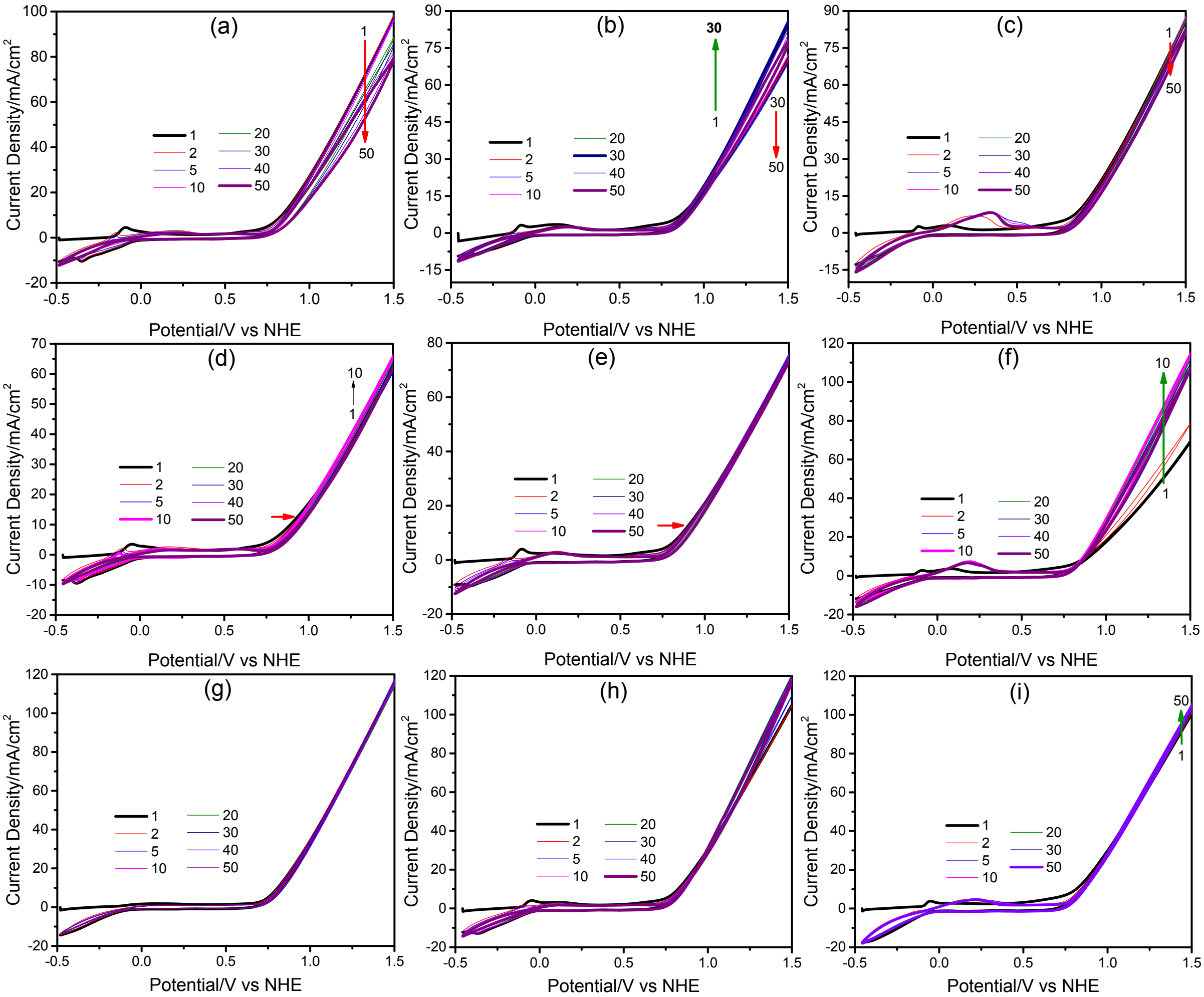

Figure S19. Selected CVs (1st, 2nd, 5th, 10th, 20th, 30th, 40th, and 50th) of 50 CV cycles (in 1M KOH solution, 50 mV/s) of the ethanol-derived GR-MnCoX-Y (a) X = 5, Y = 300, (b) X = 5, Y = 400, (c) X = 5, Y = 500, (d) X =10, Y = 300, (e) X = 10, Y = 400, (f) X = 10, Y = 500, (g) X = 25, Y = 300, (h) X = 25, Y = 400, and (i) X = 25 Y = 500.

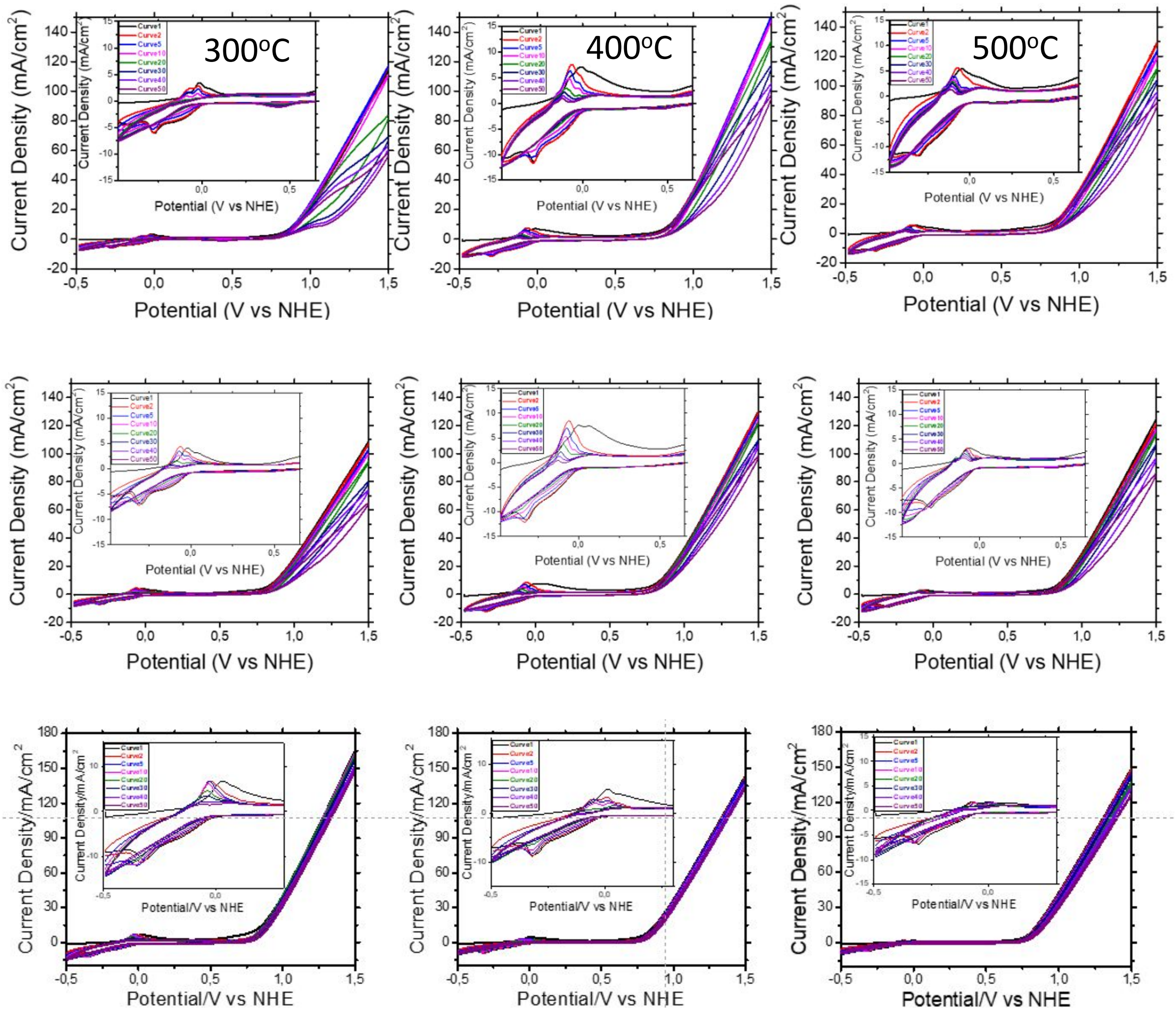

Figure S20. Selected CVs (1st, 2nd, 5th, 10th, 20th, 30th, 40th, and 50th) of 50 CV cycles (in 1 M KOH solution, 50 mV/s) of the methanol-derived GR-MnCo5-Y(1st row, left to right Y = 300, 400 and 500), GR-MnCo10-Y(2nd row, left to right Y = 300, 400 and 500), and GR-MnCo25-Y(3rd row, left to right Y = 300, 400 and 500) electrodes. Insets are redox regions.
